# Detection and remediation of organophosphorus compounds by oximate containing organogels†
†Electronic supplementary information (ESI) available: This includes experimental details and NMR data. See DOI: 10.1039/c5sc01864a



**DOI:** 10.1039/c5sc01864a

**Published:** 2015-07-10

**Authors:** Jennifer R. Hiscock, Mark R. Sambrook, Neil J. Wells, Philip A. Gale

**Affiliations:** a Chemistry , University of Southampton , Highfield , Southampton , England SO17 1BJ , UK . Email: philip.gale@soton.ac.uk ; Tel: +44 (0)23 80593332; b CBR Division , Dstl Porton Down , Salisbury , Wiltshire SP4 0JQ , UK . Email: msambrook@dstl.gov.uk ; Tel: +44 (0)1980 614301

## Abstract

A series of supramolecular diamide organogels containing a reactive compound for the remediation of organophosphorus (OP) species, in particular OP chemical warfare agents (CWAs), has been prepared in DMSO. The organogels have been found to absorb, encapsulate and decontaminate various OP CWA simulants *in situ*. At high simulant concentrations the gels also undergo a gel–sol transition releasing high local concentrations of remediation agent.

## Abstract 2

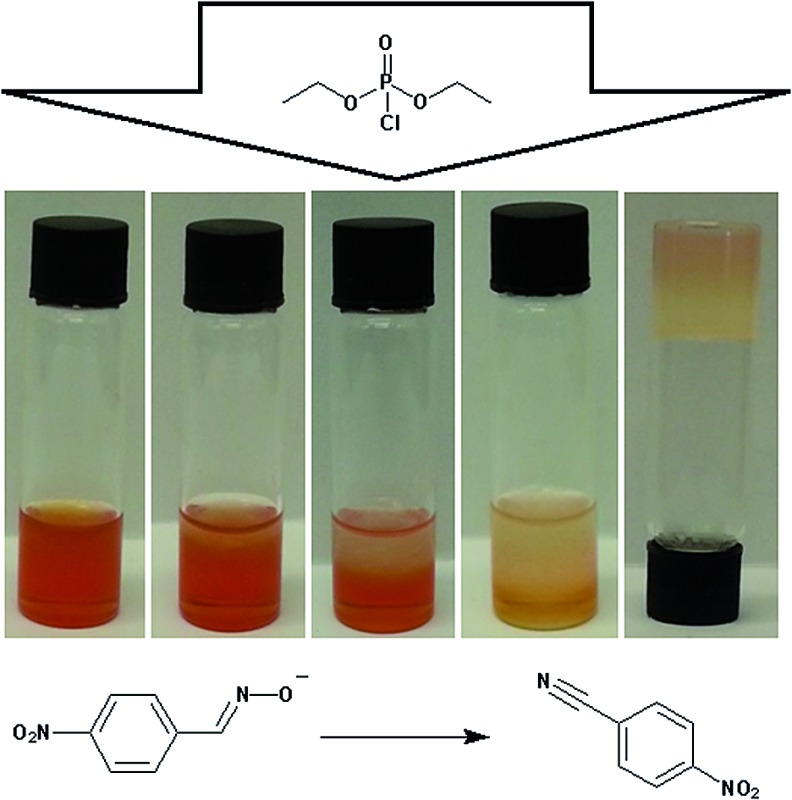
A series of supramolecular diamide organogels containing a reactive compound for the remediation of organophosphorus (OP) species, in particular OP chemical warfare agents (CWAs), has been prepared in DMSO.

## Introduction

**Fig. 1:**
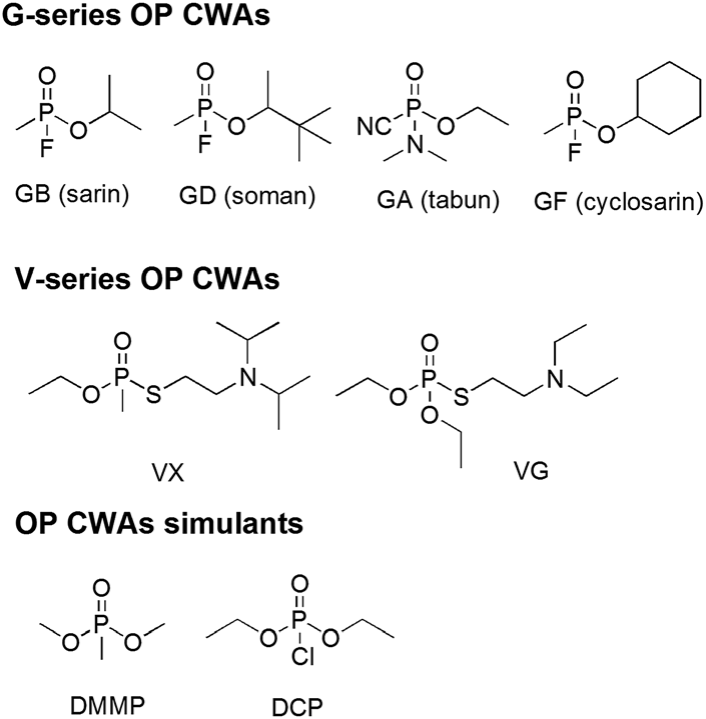
Example structures of OP CWAs and their simulants.

Organophosphorus (OP) compounds are known for their toxicity towards living organisms and include the G- and V-series chemical warfare agents (CWAs; Fig. 1). Release of OP CWAs to the environment causes indiscriminate loss of human life. The use of these compounds in terrorist attacks (Tokyo 1995)^1^ or conflict situations (Syria 2013)^2–5^ has led to a resurgence of interest in the detection and remediation of these species.^6–10^ Simulants such as DMMP and DCP (Fig. 1) are often substituted for these compounds because of the highly toxic nature and restrictions regarding the use of OP CWAs.

In previous work we have demonstrated the formation of hydrogen bonded host–guest complexes involving the coordination of GD with neutral organic receptors.^11^ Optimisation of these systems resulted in the development of a group of hydrogen bond donating molecules that were found to accelerate simulant^12^ and CWA^13^ hydrolysis.

Our recent research has focused on the development of low molecular weight gelators for the formation of supramolecular organogels that are responsive to the presence of OP compounds including CWAs. We have shown the formation of GD:gelator complexes is effective in the perturbation of hydrogen bond-mediated organogel formation.^14^ In further studies the presence of GD^15^ or simulant^16^ was found to initiate a permanent gel–sol transition through combinations of polarity effects, hydrogen bond destabilisation and gelator-simulant reactions. A simulant initiated gel–sol transition combined with a colorimetric change was also identified by Kim and co-workers. This process was shown to be reliant on reaction between simulant and organogelator.^17^ In additional research we have also been able to illustrate how the presence of an OP CWA simulant can strengthen the structure of a hydrogen bonded supramolecular organogel.^18^

Here we propose a method to immobilise and subsequently decontaminate liquid OP CWA simulants through the *in situ* formation of an organogel containing a reactive decontaminant. The remaining decontamination capacity of the gel can be monitored through colorimetric changes that result from consumption of the reactive decontaminant. The systems reported here differ to those previously reported^16^ as the gelator itself does not react with the OP CWA simulant. Instead the simulant is neutralised by the reactive decontaminant, in this case an oximate solubilised in the solvent matrix.

**Fig. 2:**
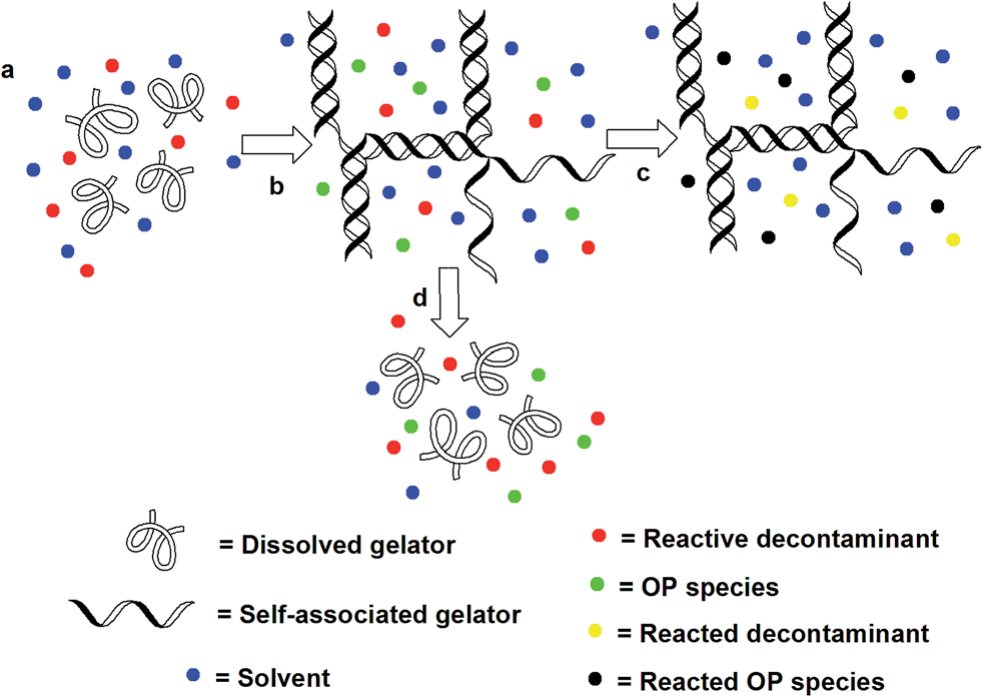
(a) Sol containing solvent, dissolved gelator and reactive decontaminant; (b) organogel formation through self-association of the gelator with the reactive decontaminant and reactive OP species held in the solution phase of the material; (c) reaction between the reactive decontaminant and simulant resulting in a colorimetric change; (d) release of reactive decontaminant upon addition of significant volumes of OP CWA simulant.

We have also investigated the behaviour of these systems in the presence of high concentrations of simulant (liquid and vapour), and shown that gel breakdown occurs with the concurrent release of oximate decontaminant. Thus depending on the concentration of gelator the gel will either absorb simulant and disclose its presence by a colour change or break down releasing decontaminant (Fig. 2).

For these experiments DMMP and DCP were chosen as appropriate simulants for OP CWAs. DCP is a highly reactive phosphate, whereas DMMP, a phosphonate, is considerably less reactive than OP CWAs. Comparison of simulant behaviour can be useful in extrapolating the behaviour expected with an OP CWA, although there are always limits to this approach. In recent work, we explored the comparative behaviour of a binary organogel^15^ to GD, DCP and DMMP addition, providing some correlation data.

## Synthesis

Five known cyclohexyldiamide compounds (**1**,^19^ **2**,^16^ **3**,^20,21^ **4** ^22^ and **5** ^21^) were synthesised by reaction of the appropriate carboxylic acid and amine with an amide coupling agent. 4-Nitrobenzaldoxime was synthesized by known literature procedures^23^ from a solution of 4-nitrobenzaldehyde and hydroxylamine hydrochloride in ethanol : water (3 : 1). Tetrabutylammonium (TBA) hydroxide was then added to a stirring methanol solution of the oxime in a 1 : 1 ratio. The methanol was removed, leaving a dark red semi-solid that was recrystallised from a solution of THF and hexane, giving TBA 4-benzaldoxime **6** as a red crystalline solid, in 99% yield.
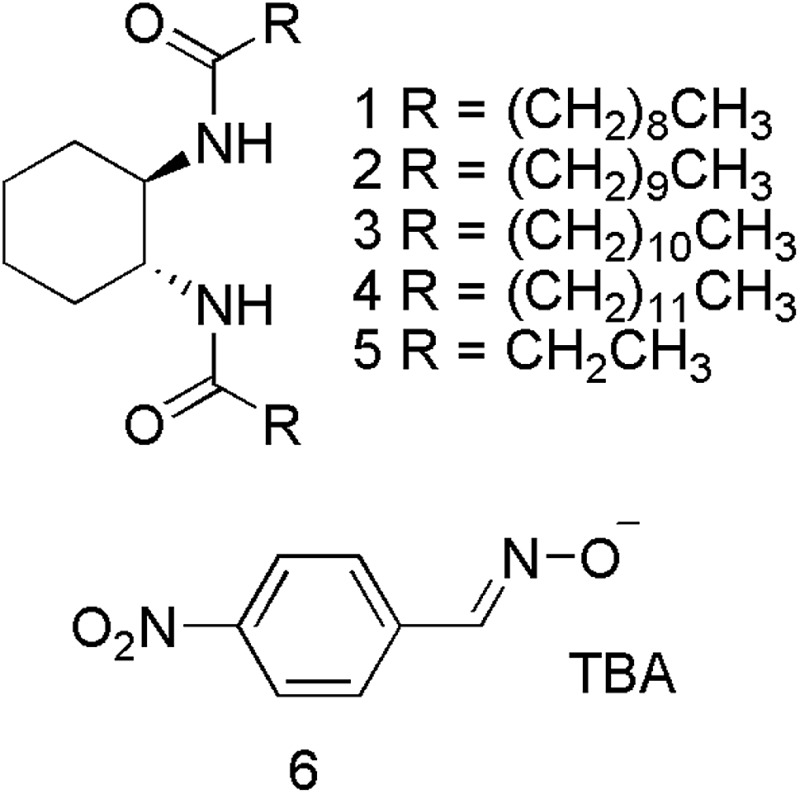


**Fig. 3:**
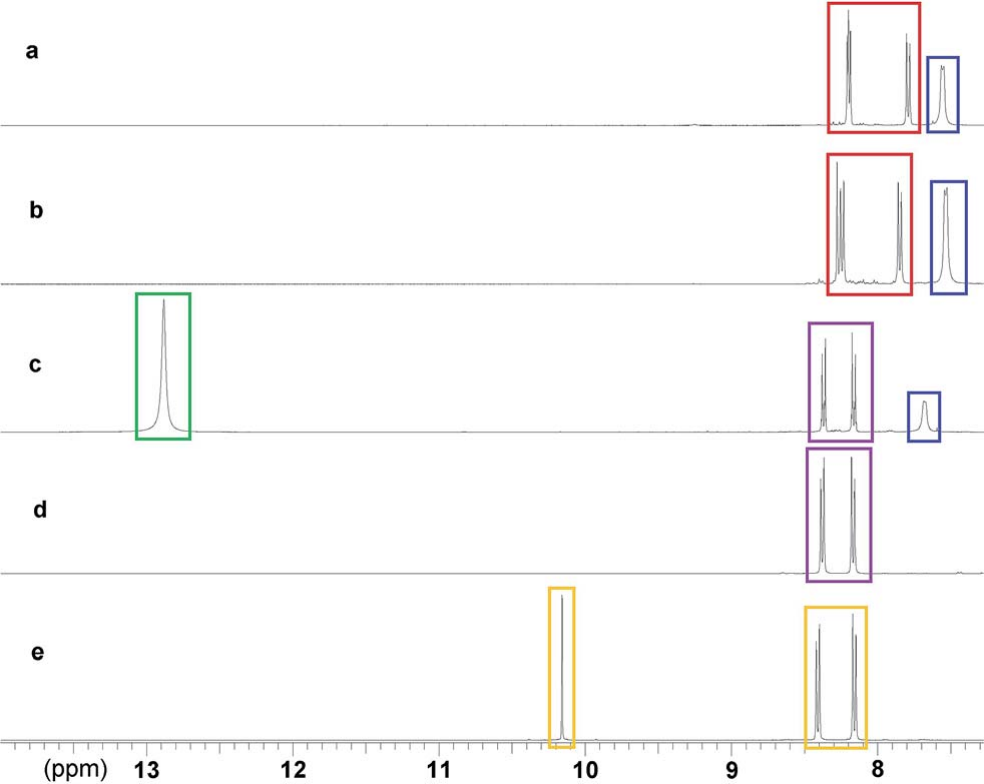
Proton NMR spectra in DMSO-*d*_6_ (0.70 mL) (a) **5** (20 mg) blue, **6** (20 mg) red; (b) **5** (20 mg) blue, **6** (20 mg) red, DMMP (0.05 mL); (c) **5** (20 mg) blue, **6** (20 mg) (which has reacted *in situ* to form **7** purple), DCP (0.05 mL) (which has reacted *in situ* to form DHP green); (d) **7** (20 mg) purple; (e) 4-nitrobenzaldhyde (20 mg) orange.

**Scheme 1:**
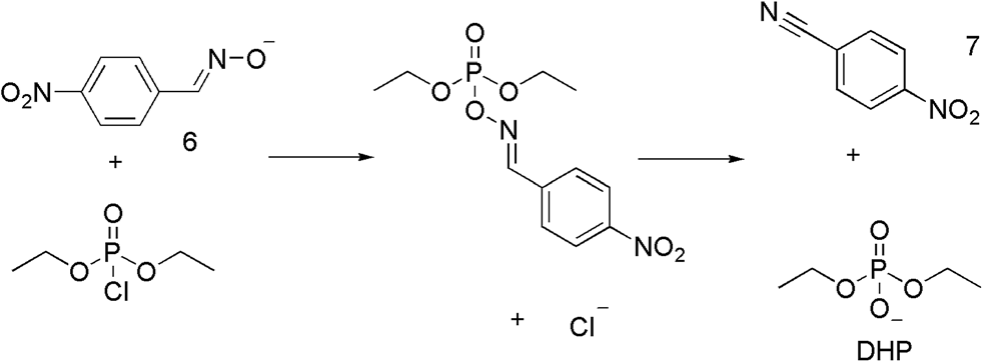
The reaction of DCP with **6**. The red colour of the oximate is lost during this reaction.

The reactive decontaminant, **6**, was incorporated into the organogels to provide the means of OP remediation. Reactions of oximates with OP CWAs and their simulants are well-known,^8,24–27^ and the parent oxime (4-nitrobenzaldoxime) is known to react with DCP and undergo a Beckman rearrangement to yield 4-nitrobenzonitrile. Confirmation of the reaction of **6** with DCP, and potentially DMMP, was sought in DMSO-*d*_6_ in the presence of **5**, which does not form an organogel and thus removes additional complexities of studying reactions within organogel substrates. The results of a series of ^1^H NMR studies are shown in Fig. 3. Fig. 3a shows the NMR spectrum of a DMSO-*d*_6_ solution of **5** and **6**, with the oximate resonances appearing at approximately 7.8 (2 × CH) and 8.2 (3 × CH multiplet) ppm. The resonance at approximately 7.6 ppm is attributed to the amide NH of **5**. These proton environments were not perturbed by the presence of DMMP, indicating the absence of any reactions under these conditions (Fig. 3b). The addition of DCP to a DMSO-*d*_6_ solution of **5** and **6** (Fig. 3c) results in the appearance of new resonance at 12.9 ppm, attributed to the OH group of diethyl hydrogen phosphate (DHP), the hydrolysis product of DCP. There is also a downfield shift of the resonances corresponding to the aromatic CH groups of **6**. The amide NH resonance of **5** is still visible at 7.7 ppm, although shifted downfield due to the increase in concentration of species present in solution that can act as hydrogen bond acceptors. This data confirms the expected Beckman rearrangement, in which DCP is rapidly consumed to yield 4-nitrobenzonitrile (Scheme 1).^26^ Comparative NMR analysis rules out the formation of 4-nitrobenzaldehdye under these conditions (Fig. 3e).

Of particular note is the intense red/orange colour of **6** that arises as a result of delocalisation in the nitro-functionalised conjugated aromatic system. The product (**7**) formed by the reaction of **6** with DCP, is colourless, thus raising the possibility of monitoring the reaction within the organogel.

## Immobilisation and remediation of OP CWA simulants

It was proposed that organogels synthesised from **1–4** in DMSO in the presence of **6** would be suitable for the immobilisation of simulants DCP and DMMP and that any remediation process within the gel would be signalled colorimetrically.

In the absence of the simulants, organogels of **1–4** were prepared by heating the gelators (typically 9.60 mg–16.00 mg) in DMSO (0.8 mL) in a sealed vial at 100 °C. The hot solutions were then allowed to cool to room temperature (approximately 21 °C) and the formation of an organogel was confirmed visually by an inversion test.^28^

Under the gelation conditions used for these experiments the minimum amount of **1** needed to form a DMSO organogel was found to be 7 mg mL^–1^ while **2–4** only required 6 mg mL^–1^ for organogel formation. Gelators **1–4** were found to gelate a DMSO solution saturated with **6** (approximately 200 mg mL^–1^) with the minimum amount of organogelator needed to gelate the equivalent amount of DMSO without **6** present.

Analogous experiments were then conducted in which a heated solution of gelator (**1–4**) and **6** in DMSO was added to a volume of DCP or DMMP (0.1–0.5 mL) and allowed to cool. Organogels were found to form in the presence of both DCP and DMMP as shown in S1 and S2.†

**Fig. 4:**
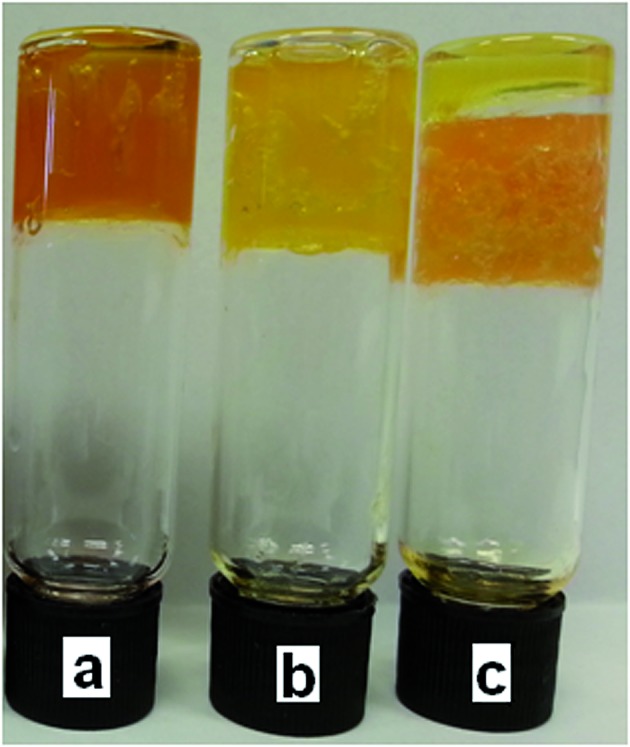
Organogels containing (a) **3** (9.6 mg), DMSO (0.8 mL), DCP (0.01 mL), **6** (80 mg); (b) **3** (9.6 mg), DMSO (0.8 mL), DCP (0.05 mL), **6** (80 mg); (c) **3** (9.6 mg), DMSO (0.8 mL), DCP (0.10 mL), **6** (80 mg).

**Fig. 5:**
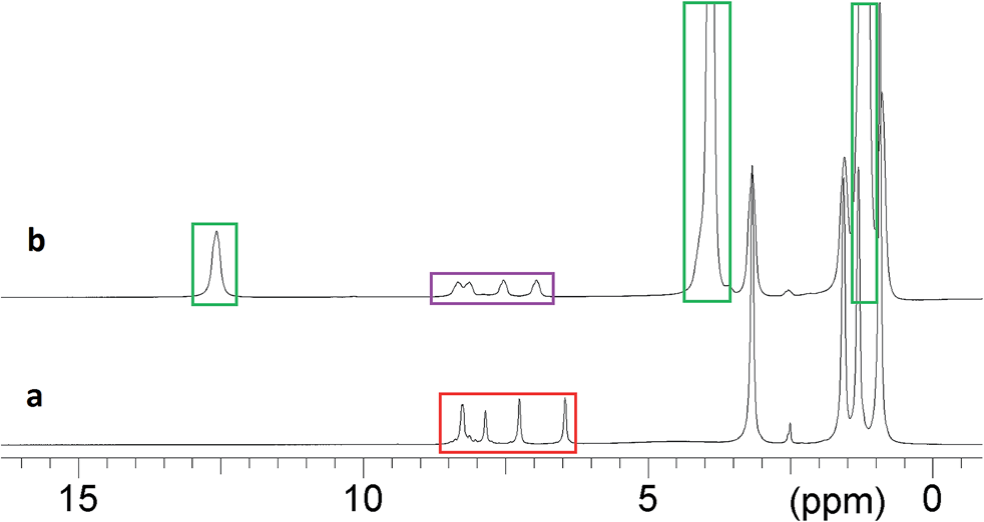
^1^H NMR spectra of organogels containing (a) **3** (11.5 mg), DMSO-*d*_6_ (0.5 mL), **6** (80 mg); (b) **3** (11.5 mg), DMSO (0.5 mL), DCP (0.1 mL), **6** (80 mg). **6** – red, *in situ* formation of **7** – purple, DCP which has reacted to form DHP – green.

In the case of DCP, addition of the hot DMSO solution resulted in an instantaneous and vigorous reaction characterised by the production of a gas, presumed to be HCl and a colour change from red to yellow (Fig. 4). As the solution cooled some of this gas was trapped in the organogel matrix, which was observed to increase proportionally with the amount of DCP present. The organogel produced by the *in situ* reaction of **6** with DCP was also analysed by NMR techniques (Fig. 5). These ^1^H NMR spectra combined with additional NMR experiments, detailed in the ESI,† provide evidence that the process shown in Scheme 1 and Fig. 3 also occurs within the gel.

**Fig. 6:**
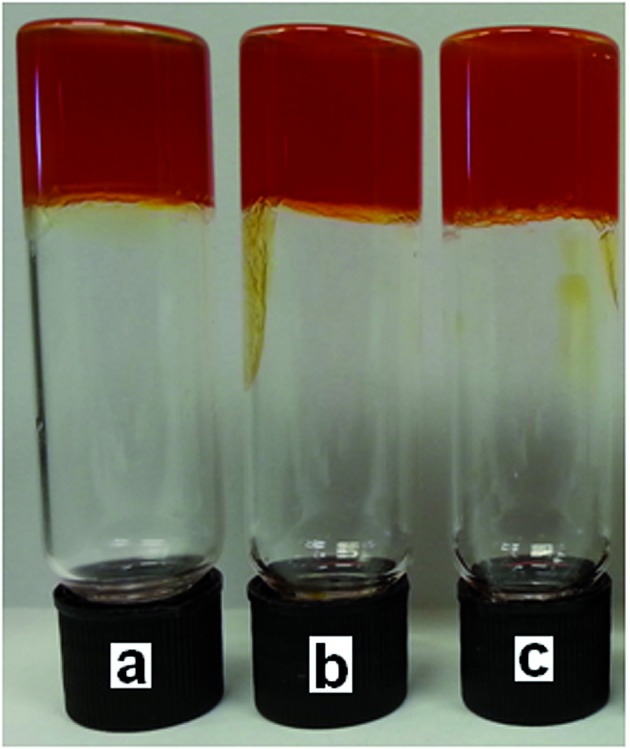
Organogels containing (a) **3** (12.0 mg), DMMP (1.00 mL), **6** (100 mg); (b) **3** (9.6 mg), DMSO (0.8 mL), DMMP (0.05 mL), **6** (80 mg); (c) **3** (9.6 mg), DMSO (0.8 mL), DMMP (0.10 mL), **6** (80 mg).

Neither a colour change nor gas evolution was observed with the organogels in the presence of DMMP indicating, as expected, that a reaction had not occurred between the simulant and **6**, as shown in Fig. 6. All of the organogels formed under these conditions with DCP or DMMP were found to remain stable over periods of time >24 h.

Gelator **4** was found to give identical results to gelator **3**, while **1** and **2** exhibit lower maximum % volumes of DCP addition for full organogel formation. The longer alkyl chains of **3** and **4** may be responsible for the enhanced stability of the organogels under these conditions.

For gelator **3** the maximum % volume of DCP addition to the organogelator/oxime mixtures allowing full organogel formation was between 27 and 33%. In the presence of higher % volumes of DCP a yellow solution or partial organogel was formed. Failure to produce a gel may be due to one or a combination of; increasing solvent polarity, a change in gelator solubility and an increased number of compounds capable of acting as hydrogen bond acceptors contributing to destruction of the solid hydrogen bonded gelator matrix. Increasing the amount of organogelator present in the solution had no effect on the maximum % volume of DCP addition allowing full organogel stability, however, increasing the amount of **6** present from 80 to 160 mg does lower the maximum % volume of DCP to between 20 and 27%.

## Sensing liquid and vapour OP simulants

**Fig. 7:**
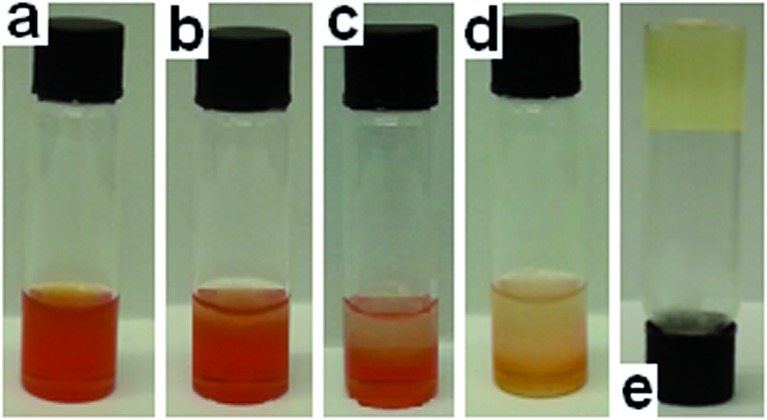
Organogel containing (a) DMSO (1 mL); **3** (12 mg mL^–1^) and **6** (10 mg mL^–1^); (b) 60 seconds after DCP (0.1 mL) addition; (c) 25 min after DCP addition; (d) 60 min after DCP addition; (e) 22 h after DCP addition. Temperature maintained at approximately 21 °C.

We wished to establish whether gels containing compound **6** could be used as self-disclosing remediation materials. To test this the surface of the organogel was exposed to OP CWA simulant liquid and vapour. Organogels were prepared in DMSO (1.0 mL) as illustrated in Fig. 7. DCP was then added to the surface of the material and the vial sealed. The evolution of gas (presumed to be HCl) from the surface of the organogel and discolouration of the organogel was observed. The red-to-yellow colour change was found to initiate at the surface of the organogel and proceed to the bottom of the vial over time as the DCP/reaction products diffused throughout the material. The rate of the colour change process increased as the amount of **6** present in the organogel decreased. In all cases the total volume of DCP was absorbed into the organogel matrix which then remained stable for periods of time >24 h.

**Fig. 8:**
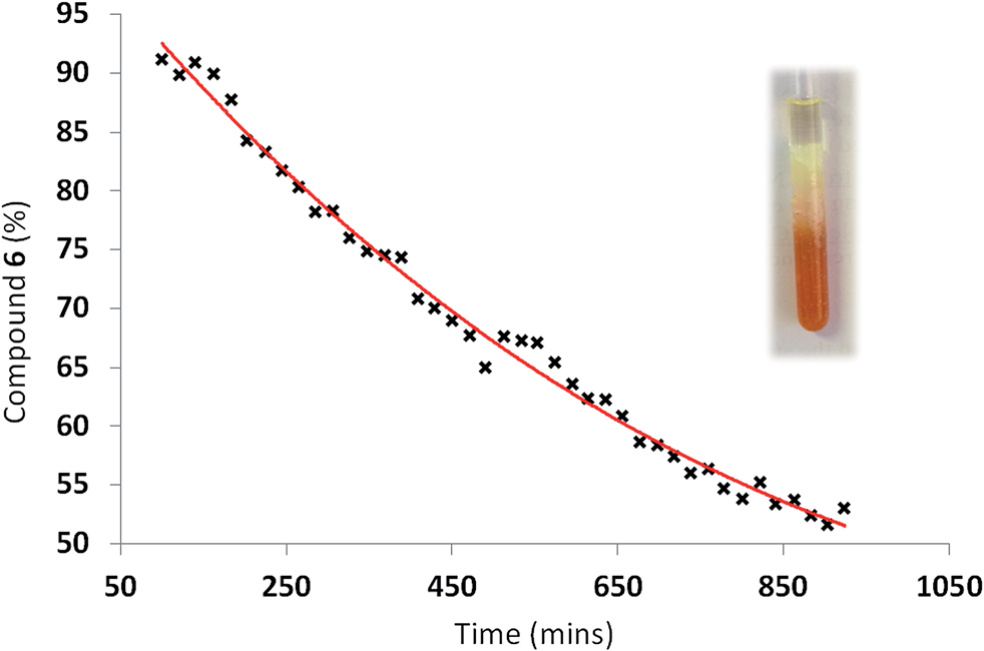
Effects of DCP (0.1 mL) addition to the surface of an organogel (DMSO-*d*_6_ (0.5 mL), **6** (80 mg) and **3** (11.5 mg)) with respect to time. The photograph shows the end point of the ^1^H NMR experiment.

The course of the reaction within the organogel during this process was also monitored by ^1^H NMR. An organogel was formed from a warmed solution of DMSO-*d*_6_ (0.5 mL), **6** (80 mg) and **3** (11.5 mg). This mixture was transferred into an NMR tube (5 mm) and allowed to cool to room temperature upon which the expected gel formation was observed to occur. DCP (0.1 mL) was then added to the NMR tube forming a distinct volume of liquid on the surface of the gel, and the NMR tube was then sealed. The surface of the gel was approximately 4 mm above the top of the NMR aperture, which was approximately 23 mm in length. Reaction of DCP with **6** was then observed as the DCP diffused through the gel and into the aperture observable by the spectrometer. The signals corresponding to reacted and unreacted **6** were then integrated and used to calculate the percentage of unreacted **6** present in the gel volume presenting the NMR aperture (Fig. 8).

**Fig. 9:**
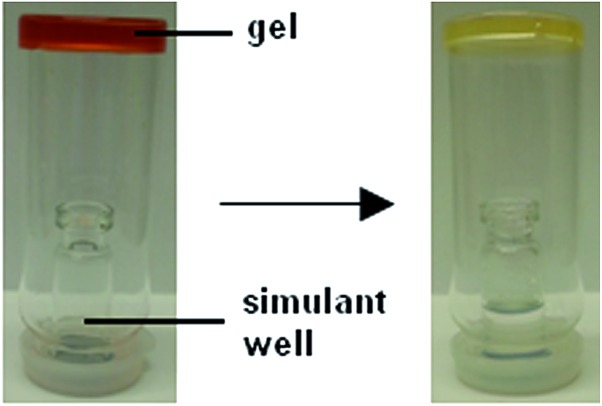
Experimental setup for testing the effects of DCP vapours on an organogel; (left) experimental setup without the presence of DCP in the simulant well; (right) effects of DCP addition to the simulant well.

This gel was also tested against DCP vapour as shown in Fig. 9. An inverted vial containing organogel was placed over a simulant well. After DCP (0.1 mL) was added to the simulant well the vials were sealed and left for 23 h. The DCP vapours were produced by evaporation of the stock solution from the simulant well. The organogels used for this experiment contained **3** (12 mg mL^–1^), DMSO (1 mL) and varying amounts of **6**. All the samples tested were all found to remain stable over the 23 h. The colour intensity of gels containing **6** was found to change on exposure to DCP vapours. The most noticeable colour changes were noted with the organogels that contained the least amount of **6**.

## Remediation solution release

**Fig. 10:**
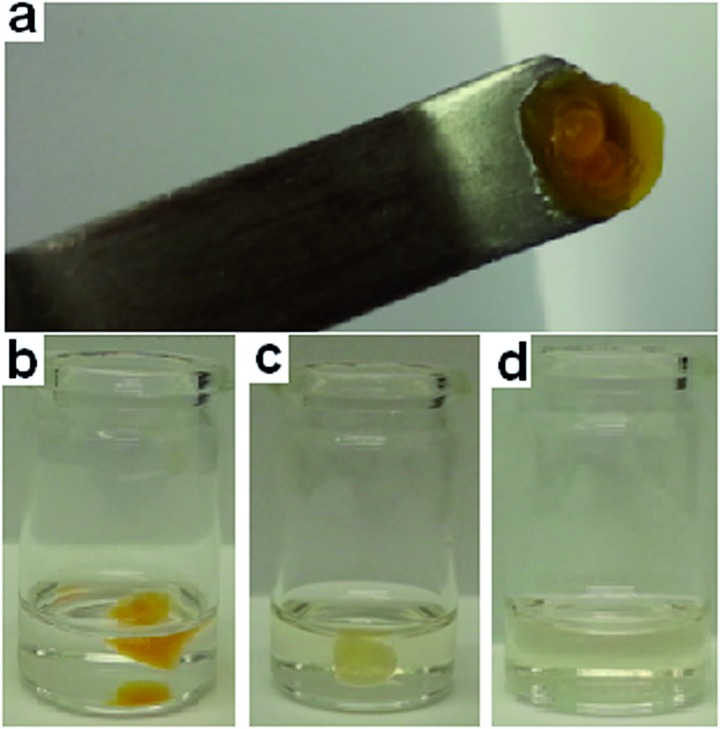
(a) Organogel tab; (b) 60 seconds after organogel addition to DCP; (c) 15 min after organogel addition to DCP; (d) 2.25 h after organogel addition to DCP.

Although the organogels tested are stable to DCP addition/adsorption, there is a limit which if reached initiates a secondary gel–sol phase change response. This results in the release of the oximate containing remediation solution from the organogel matrix. The gel–sol transition (as illustrated in Fig. 10) is most likely due to one or more of the following factors: a change in solvent polarity, increased solvent volume and/or hydrogen bond destabilisation. In this example organogel tabs were created by spotting a hot solution of DMSO (0.1 mL), **3** (1.2 mg) and **6** (10 mg) onto a glass plate. When solidified this organogel tab was placed into DCP (1 mL). Over time the tab was found to dissolve and change colour releasing the remediation solution contained within the solid structure into the surrounding solution.

## Conclusions

We have developed a new class of responsive supramolecular organogel for the remediation of reactive OPs such as CWAs. These materials are formed from unreactive supramolecular gelators, combined with a DMSO/oximate remediation solution. We have shown that these gels can used to immobilise and decontaminate liquid contamination. Secondly, they can be used as sensing materials to disclose the presence of CWA liquid and vapour hazards. Thirdly, the collapse of these organogel in the presence of large volume of OP CWA could be used to release local, high concentrations of oximate decontaminant. We believe these materials demonstrate a new approach to the detection and radiation of organophosphorus compounds.
