# Supplementary material for: Detection and remediation of organophosphorus compounds by oximate containing organogels
Source: Chem Sci. 2015 Jul 10;6(10):5680–4. doi: 10.1039/c5sc01864a (PMC5527500; doi:10.1039/c5sc01864a)
Supplement: Supplementary file 1 [file SC-006-C5SC01864A-s001.pdf]

Electronic Supplementary Material (ESI) for Chemical Science.
This journal is © The Royal Society of Chemistry 2015

Supramolecular gels for the remediation of organophosphorus compounds

Jennifer R. Hiscock[a], Mark R. Sambrook[b], Neil J. Wells[a] and Philip A. Gale*[a]

[a] School of Chemistry, University of Southampton, Southampton, UK SO17 1BJ. E-mail: philip.gale@soton.ac.uk; Fax: +44 (0)23 8059 6805; Tel: +44 (0)23 8059 3322.

[b] CBR Division, Dstl Porton Down, Salisbury, Wiltshire, SP4 0JQ. Tel: +44 1980 614301; Email: msambrook@dstl.gov.uk.

Electronic Supplementary Information

Information referenced in the main manuscript

S1 Combinations of **3**, solvent, simulant and **6** that were evaluated for *in situ* gel formation.

Gel formed	Gelator (mg)	Simulant (mL)		DMSO (mL)	**6** (mg)
**Yes**	9.6	DMMP	0.10	0.8	80
**Yes**	9.6	DMMP	0.05	0.8	80
**Yes**	9.6	DMMP	0.01	0.8	80
**Yes**	9.6	DCP	0.01	0.8	80
**Yes**	9.6	DCP	0.20	0.8	80
**Yes**	9.6	DCP	0.30	0.8	80
**No**	9.6	DCP	0.40	0.8	80
**No**	9.6	DCP	0.50	0.8	80
**Yes**	9.6	DCP	0.10	0.8	160
**Yes**	9.6	DCP	0.20	0.8	160
**No**	9.6	DCP	0.30	0.8	160
**No**	9.6	DCP	0.40	0.8	160
**No**	9.6	DCP	0.50	0.8	160
**Yes**	16.0	DCP	0.10	0.8	80
**Yes**	16.0	DCP	0.20	0.8	80
**Yes**	16.0	DCP	0.30	0.8	80
**No**	16.0	DCP	0.40	0.8	80
**No**	16.0	DCP	0.50	0.8	80
**Yes**	12.0	DMMP	1.00	0.0	100

S2 Various combinations of **1**, **2** and **4**, solvent, simulant and **6** that were evaluated for *in situ* gel formation.

Gelator	Organogel formed	Gelator (mg)	Simulant (mL)		DMSO (mL)	**6** (mg)
**1**	**Yes**	12.0	DMMP	1.0	0.0	100
**1**	**Yes**	9.6	DCP	0.1	0.8	80
**1**	**Yes**	9.6	DCP	0.2	0.8	80
**1**	**No**	9.6	DCP	0.3	0.8	80
**1**	**No**	9.6	DCP	0.4	0.8	80
**1**	**No**	9.6	DCP	0.5	0.8	80
**2**	**Yes**	12.0	DMMP	1.0	0.0	100
**2**	**Yes**	9.6	DCP	0.1	0.8	80
**2**	**Yes**	9.6	DCP	0.2	0.8	80
**2**	**No**	9.6	DCP	0.3	0.8	80
**2**	**No**	9.6	DCP	0.4	0.8	80
**2**	**No**	9.6	DCP	0.5	0.8	80
**4**	**Yes**	12.0	DMMP	1.0	0.0	100
**4**	**Yes**	9.6	DCP	0.1	0.8	80
**4**	**Yes**	9.6	DCP	0.2	0.8	80
**4**	**Yes**	9.6	DCP	0.3	0.8	80
**4**	**No**	9.6	DCP	0.4	0.8	80
**4**	**No**	9.6	DCP	0.5	0.8	80

Experimental

General remarks: All reactions were performed under slight positive pressure of nitrogen using oven-dried glassware. All solvents and starting materials were purchased from chemical stores where available. Commercial grade reagents have been used without further purification. ^{1}H NMR (500 MHz) and ^{13}C{^{1}H} NMR (125 MHz) were determined on a Bruker AV III HD 500 spectrometer , ^{1}H NMR (400 MHz), ^{31}P{^{1}H} NMR (162 MHz) and ^{13}C{^{1}H} NMR (100 MHz) were determined on a Bruker AV II 400 or AV III HD 400 spectrometer with the chemical shifts reported in parts per million (ppm), calibrated to the centre of the solvent peak set. Some ^{13}C peaks are missing due to overlapping signals. The following abbreviations are used for spin multiplicity: s = singlet, d = doublet, t = triplet, m = multiplet, br = broad. Infrared (IR) spectra were recorded on a Matterson Satellite (ATR), and reported in wavenumbers (cm^{-1}). Low resolution mass spectra were recorded on a Waters Acquity UHPLC-MS system. High resolution mass spectra were recorded on a Bruker Maxis ESI-TOF system by the mass spectrometry service at the University of Southampton. Melting points were recorded in open capillaries on a Gallenkamp melting point apparatus and are uncorrected.

Compound 2 was synthesised in accordance with the literature procedure given by Gale and Co-workers.[1] ^{1}H NMR (400 MHz, $CDCl_3$): δ: 0.87 (t, J = 6.08 Hz, 6H), 1.25 (br s, 32H), 1.56 (br s, 4H), 1.74 (d, J = 8.16 Hz, 2H), 2.02 (d, J = 12.08 Hz, 2H), 2.10 (dd, J_1 = 11.60 Hz, J_2 = 6.96 Hz, 4H), 3.65 (br s, 2H), 5.93 (d, J = 4.40 Hz, 2H, amide NH).

Compound 1 was synthesised by the same general method given by Gale and Co-workers.[1] Proton NMR spectrum was found to match that previously published by Sato and co-workers.[2]

Compound 3 was synthesised by the same general method given by Gale and Co-workers.[1] Proton NMR spectrum was found to match that previously published by Esch and co-workers.[3]

Compound 4 was synthesised by the same general method given by Gale and Co-workers.[1] Although previously published by Kimura, Shirai and co-workers[4] full characterisation of this compound was performed. [1] Proton NMR spectrum was found to match that previously published by Gale and co-workers.[1]

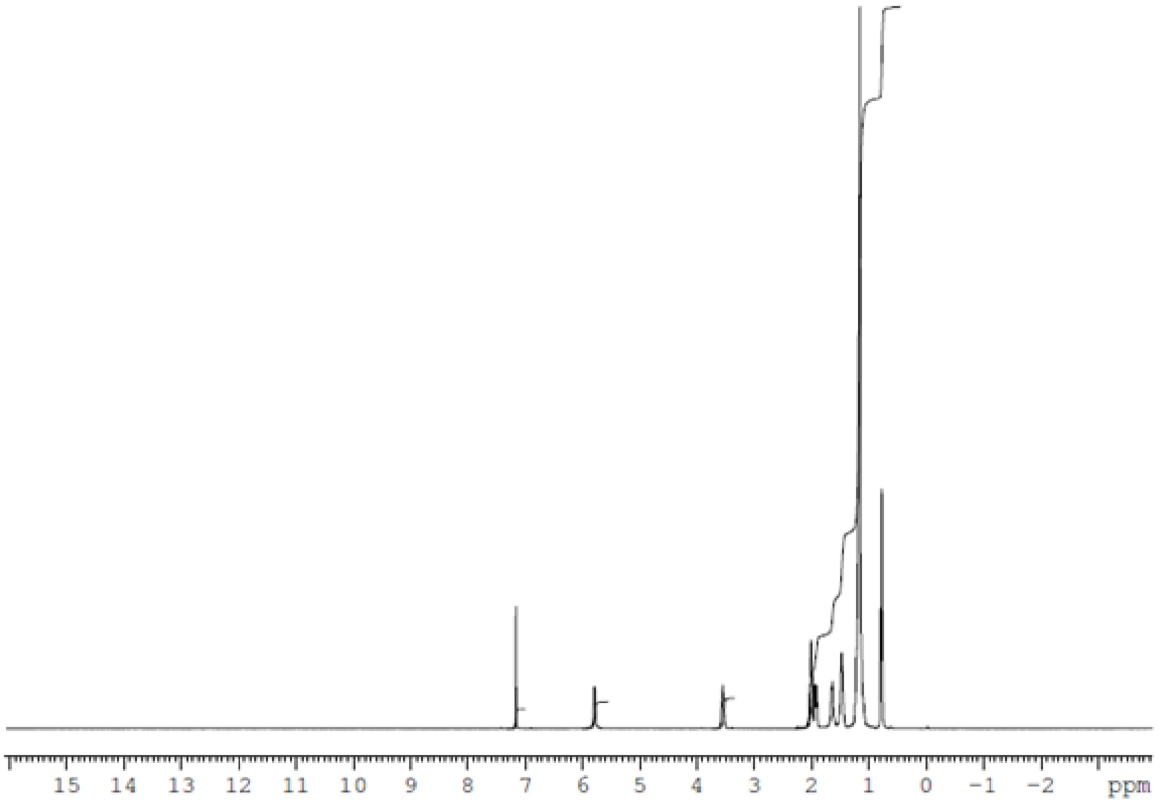

S3 ^{1}H NMR spectrum of compound **1** in $CDCl_3$.

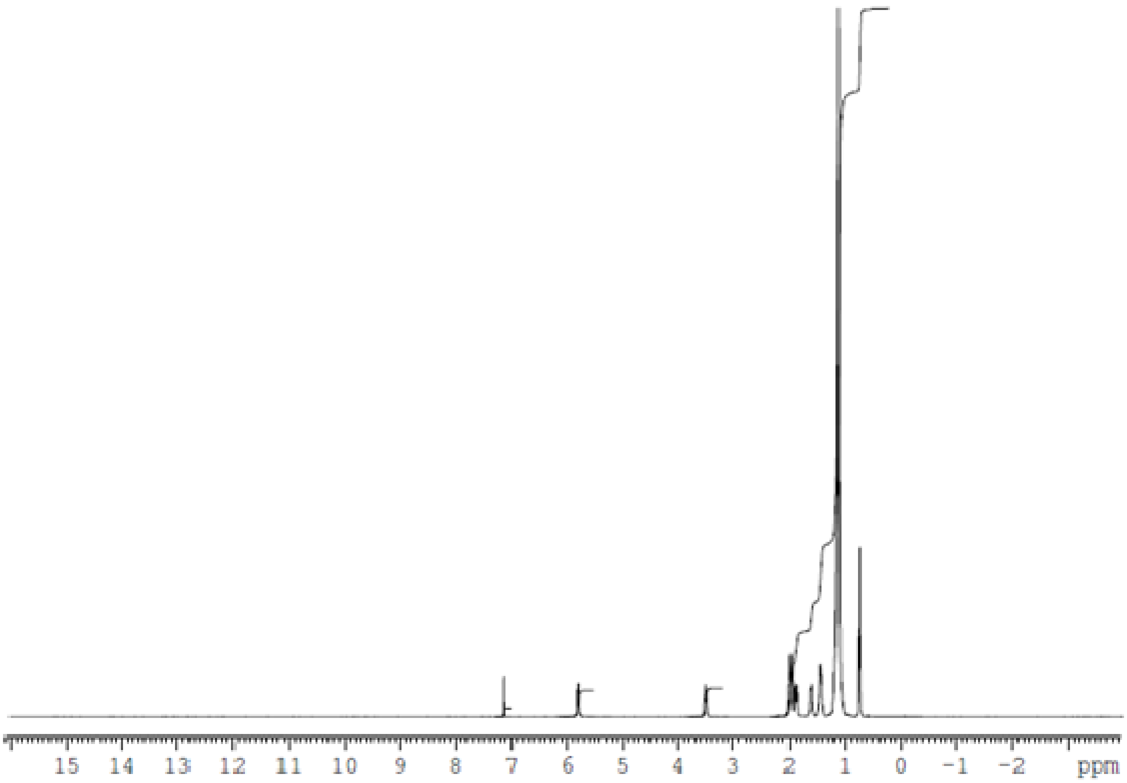

S4 ^{1}H NMR spectrum of compound **2** in $CDCl_3$.

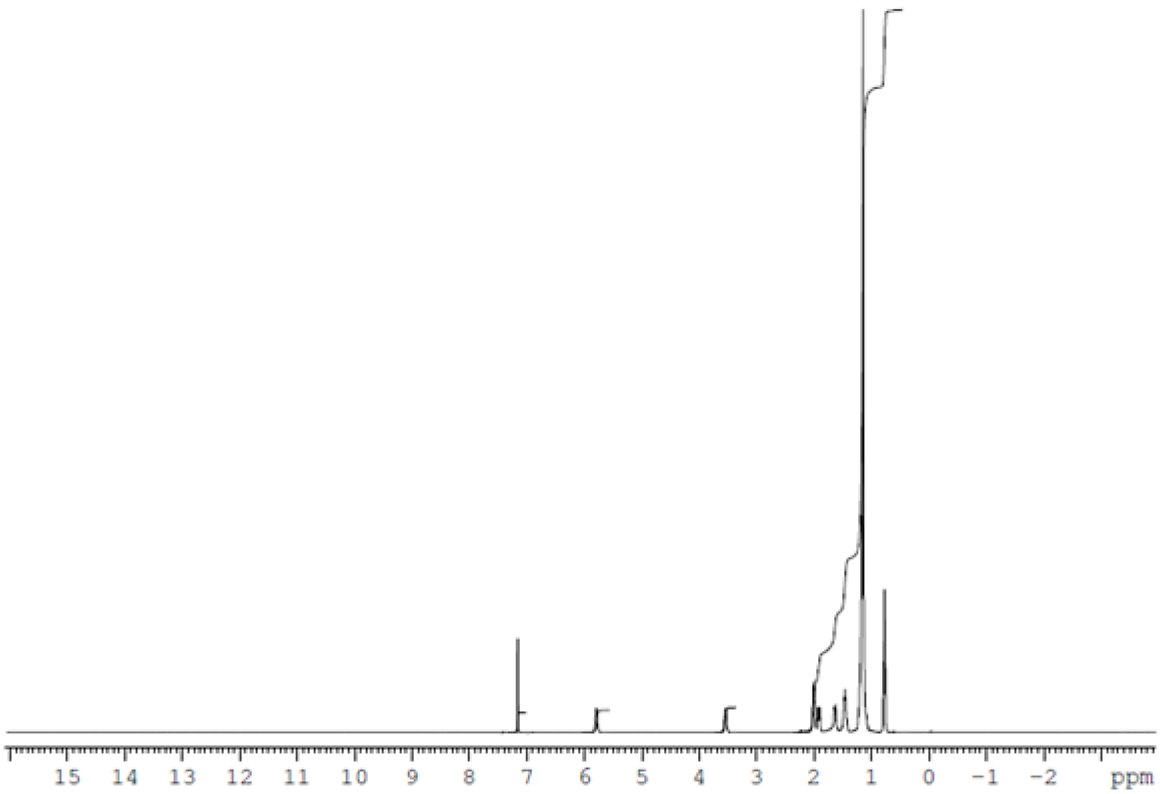

S5 ^{1}H NMR spectrum of compound **3** in $CDCl_3$.

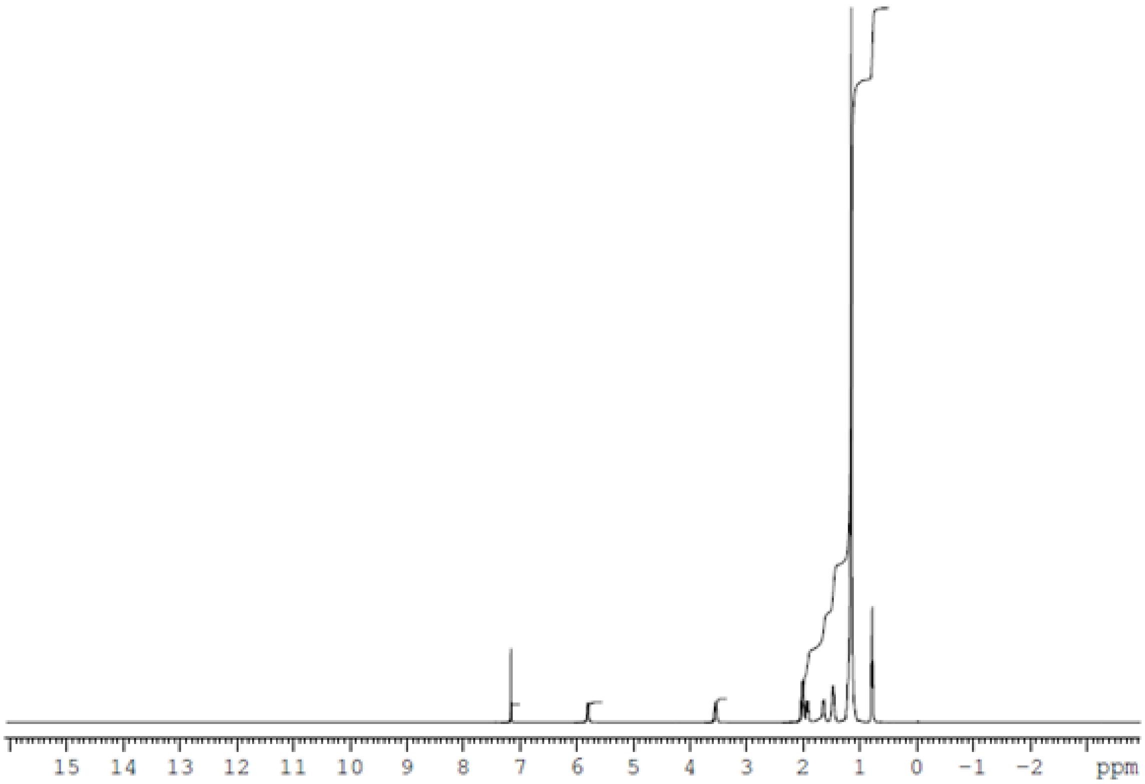

S6 ^{1}H NMR spectrum of compound **4** in $CDCl_3$.

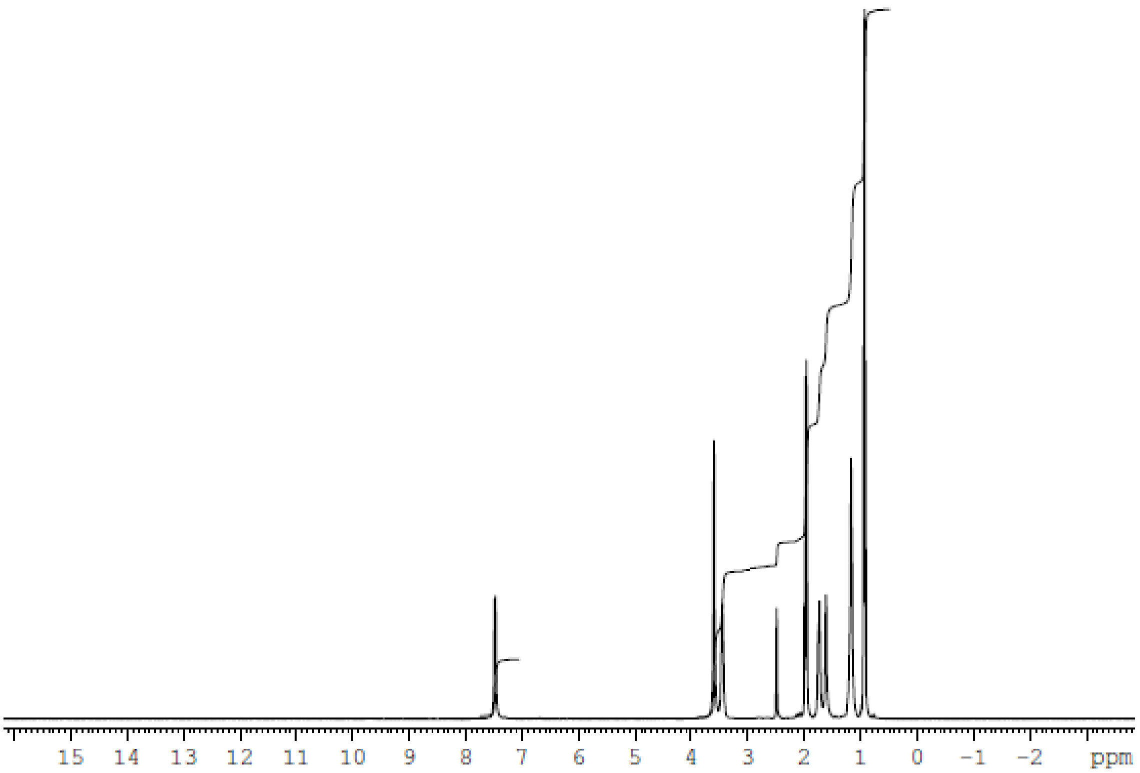

S7 ^{1}H NMR spectrum of compound **5** in DMSO-d_6.

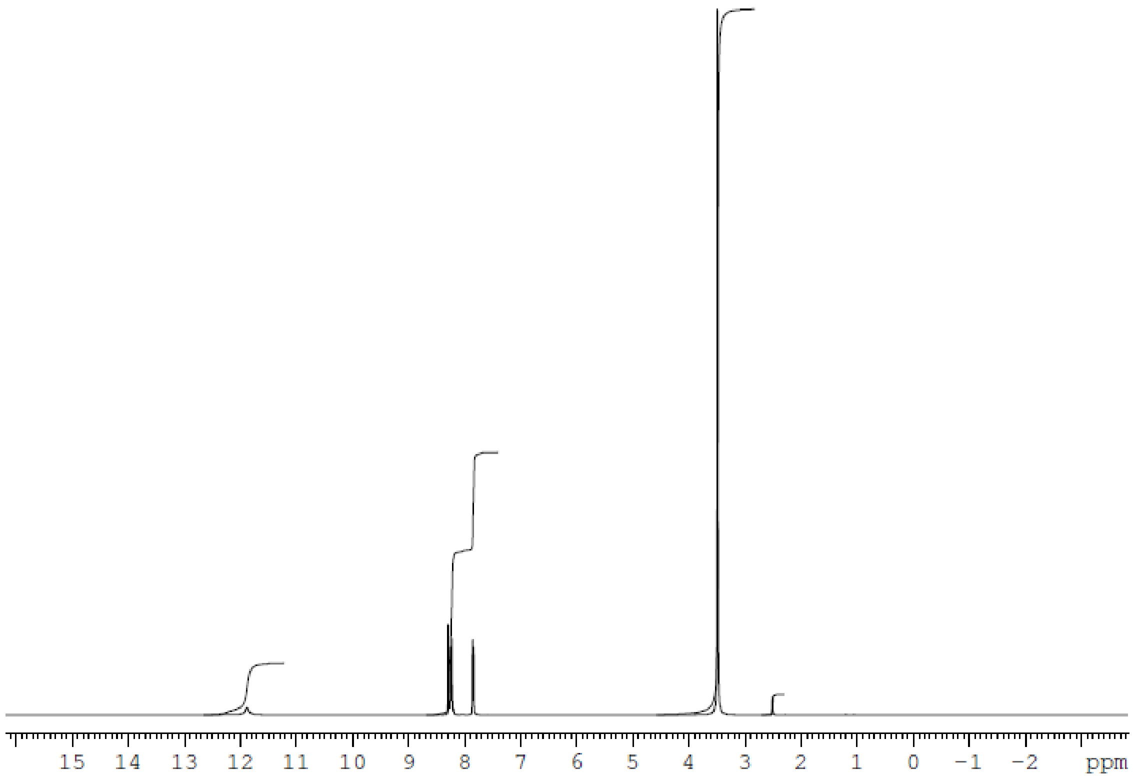

S8 ^{1}H NMR spectrum of 4-nitrobenzoxime in DMSO-d_6.

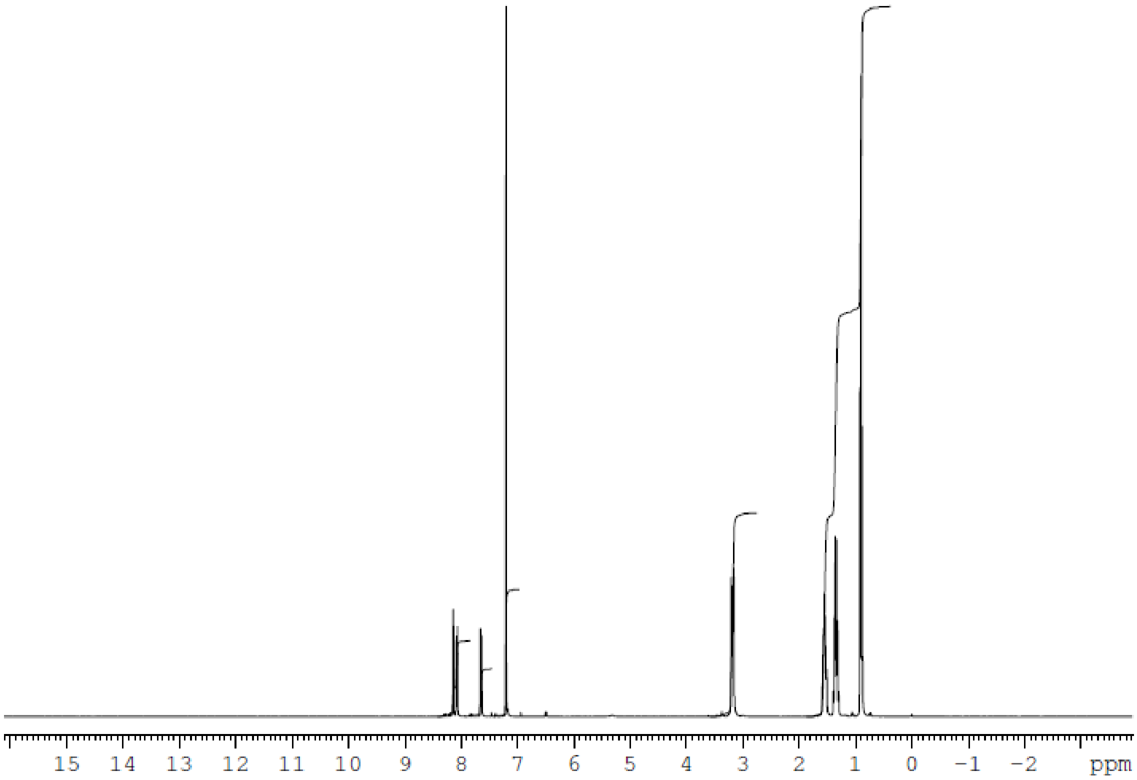

S9 ^{1}H NMR spectrum of compound **6** in $CDCl_3$.

S10 Amount of gelator, compounds **1-4** and **6** (mmol) and added phosphonate (mmol).

**Gelator**	**Amount of gelator in 1 mL of gel (mmol)**								
	25 mg	20 mg	15 mg	10 mg	5.0 mg	4.0 mg	3.0 mg	2.5 mg	2.0 mg
**1**	0.059	0.047	0.035	0.024	0.012	0.009	0.007	0.006	0.005
**2**	0.055	0.044	0.033	0.022	0.011	0.009	0.007	0.006	0.004
**3**	0.052	0.042	0.031	0.021	0.010	0.008	0.006	0.005	0.004
**4**	0.049	0.039	0.029	0.020	0.010	0.008	0.006	0.005	0.004
**Oximate**	**Amount of oximate in gel samples (mmol)**								
	200 mg	160 mg	100 mg	80 mg	40 mg	20 mg	10 mg		
**5**	0.491	0.393	0.245	0.196	0.098	0.049	0.025		
**Phosphonate**	**Amount of phosphonate added (mmol)**								
	1.000 mL	0.100 mL	0.050 mL	0.010 mL	0.001 mL				
DCP	6.955	0.695	0.348	0.070	0.007				
DMMP	9.228	0.923	0.461	0.092	0.009				

S11 Formation of gels with compound **1**, all experiments were performed with 1 mL of the solvent stated.

**Concentration of gelator (mg/mL)**	**Solvent**		
	**DMMP**	**DCP**	**DMSO**
40.0	NA	No Gel	NA
20.0	Gel	NA	Gel
10.0	Gel	NA	Gel
7.0	NA	NA	Gel
6.0	NA	NA	Partial Gel
5.0	Gel	NA	Partial Gel
4.0	Partial Gel	NA	NA
3.0	Partial Gel	NA	NA
2.5	No Gel	NA	NA

S12 Formation of gels with compound **2**, all experiments were performed with 1 mL of the solvent stated.

**Concentration of gelator (mg/mL)**	**Solvent**		
	**DMMP**	**DCP**	**DMSO**
40.0	NA	No Gel	NA
20.0	Gel	NA	Gel
10.0	Gel	NA	Gel
7.0	NA	NA	NA
6.0	NA	NA	Gel
5.0	Gel	NA	Partial Gel
4.0	Gel	NA	NA
3.0	Gel	NA	NA
2.5	Partial Gel	NA	NA

S13: Formation of gels with compound **3**, all experiments were performed with 1 mL of the solvent stated.

Concentration of gelator (mg/mL)	Solvent		
	DMMP	DCP	DMSO
40.0	NA	Partial Gel/ Precipitation	NA
20.0	Gel	Partial Gel	Gel
10.0	Gel	NA	Gel
7.0	NA	NA	NA
6.0	NA	NA	Gel
5.0	Gel	NA	Partial Gel
4.0	Gel	NA	NA
3.0	Gel	NA	NA
2.5	Partial Gel	NA	NA

S14: Formation of gels with compound **4**, all experiments were performed with 1 mL of the solvent stated.

Concentration of gelator (mg/mL)	Solvent		
	DMMP	DCP	DMSO
40.0	NA	No Gel	NA
20.0	Gel	NA	Gel
10.0	Gel	NA	Gel
7.0	NA	NA	NA
6.0	NA	NA	Gel
5.0	Gel	NA	Partial Gel
4.0	Gel	NA	NA
3.0	Gel	NA	NA
2.5	Partial Gel	NA	NA

S15 Formation of gels with compounds **1**-**4**, various mixtures of gelator, solvent and compound **6** were heated to approximately 100 ºC until all the solid had dissolved and added to aliquots of simulant and allowed to cool to room temperature, apart from the cases where no DMSO was used. In these cases the mixtures of gelator and oximate were heated directly in the simulant until all the solid had dissolved at which time the solutions were allowed to cool to room temperature. Solutions containing compound **6** are dark red in colour until DCP is added. Orange gels did lighten over time to become yellow in colour but did remain stable. Original colour change observed 15 mins after gelation solution was removed from the heat source.

Gelator	Gelator Addition (mg)	Simulant	Simulant Addition (mL)	DMSO Addition (mL)	Compound **6** (mg)	Observation
**3**	9.60	DMMP	0.10	0.80	80.00	Red gel
**3**	9.60	DMMP	0.05	0.80	80.00	Red gel
**3**	9.60	DMMP	0.01	0.80	80.00	Red gel
**3**	9.60	DCP	0.10	0.80	80.00	Yellow gel containing gas bubbles
**3**	9.60	DCP	0.05	0.80	80.00	Yellow gel containing gas bubbles
**3**	9.60	DCP	0.01	0.80	80.00	Yellow gel containing gas bubbles
**3**	9.60	DCP	0.20	0.80	80.00	Yellow gel containing gas bubbles
**3**	9.60	DCP	0.30	0.80	80.00	Yellow gel containing gas bubbles
**3**	9.60	DCP	0.40	0.80	80.00	Yellow solution
**3**	9.60	DCP	0.50	0.80	80.00	Yellow solution
**3**	9.60	DCP	0.10	0.80	160.00	Orange gel
**3**	9.60	DCP	0.20	0.80	160.00	Orange gel
**3**	9.60	DCP	0.30	0.80	160.00	Partial yellow gel
**3**	9.60	DCP	0.40	0.80	160.00	Yellow solution
**3**	9.60	DCP	0.50	0.80	160.00	Yellow solution
**3**	16.00	DCP	0.10	0.80	80.00	Yellow gel containing gas bubbles
**3**	16.00	DCP	0.20	0.80	80.00	Yellow gel containing gas bubbles
**3**	16.00	DCP	0.30	0.80	80.00	Yellow gel containing gas bubbles
**3**	16.00	DCP	0.40	0.80	80.00	Partial yellow gel
**3**	16.00	DCP	0.50	0.80	80.00	Yellow solution
**3**	12.00	DCP	1.00	0.00	100.00	Yellow solution
**3**	12.00	DMMP	1.00	0.00	100.00	Red gel
**3**	12.00	DCP	1.00	0.00	200.00	Yellow solution
**3**	50.00	DCP	1.00	0.00	200.00	Yellow viscous solution
**1**	12.00	DMMP	1.00	0.00	100.00	Red gel
**1**	9.60	DCP	0.10	0.80	80.00	Yellow gel containing gas bubbles
**1**	9.60	DCP	0.20	0.80	80.00	Yellow gel containing gas bubbles
**1**	9.60	DCP	0.30	0.80	80.00	Yellow solution
**1**	9.60	DCP	0.40	0.80	80.00	Yellow solution
**1**	9.60	DCP	0.50	0.80	80.00	Yellow solution
**2**	12.00	DMMP	1.00	0.00	100.00	Red gel
**2**	9.60	DCP	0.10	0.80	80.00	Yellow gel containing gas bubbles
**2**	9.60	DCP	0.20	0.80	80.00	Yellow gel containing gas bubbles
**2**	9.60	DCP	0.30	0.80	80.00	Yellow solution
**2**	9.60	DCP	0.40	0.80	80.00	Yellow solution
**2**	9.60	DCP	0.50	0.80	80.00	Yellow solution
**4**	12.00	DMMP	1.00	0.00	100.00	Red gel
**4**	9.60	DCP	0.10	0.80	80.00	Yellow gel containing gas bubbles
**4**	9.60	DCP	0.20	0.80	80.00	Yellow gel containing gas bubbles
**4**	9.60	DCP	0.30	0.80	80.00	Yellow gel containing gas bubbles
**4**	9.60	DCP	0.40	0.80	80.00	Yellow solution
**4**	9.60	DCP	0.50	0.80	80.00	Yellow solution

S16 Formation of gels with compounds **1**-**4**, various mixtures of gelator, solvent and compound **6** were heated to approximately 100 ºC until all the solid had dissolved and added to aliquots of simulant and allowed to cool to room temperature. DCP was then added to the surface of the gel and allowed to diffuse into the gel structure leaving a colourless gel, as in Figure 3.

Gelator	Gelator Amount (mg)	Simulant	Simulant Amount (mL)	DMSO Amount (mL)	**6** Amount (mg)
**1**	12.00	DCP	0.10	1.00	160.00
**2**	12.00	DCP	0.10	1.00	160.00
**3**	12.00	DCP	0.10	1.00	160.00
**4**	12.00	DCP	0.10	1.00	160.00
**3**	12.00	DCP	0.10	1.00	0.00
**3**	12.00	DCP	0.10	1.00	10.00
**3**	12.00	DCP	0.10	1.00	20.00
**3**	12.00	DCP	0.10	1.00	40.00
**3**	12.00	DCP	0.10	1.00	80.00
**3**	20.00	DCP	0.10	1.00	160.00

S17 Formation of gels with compounds **1**-**4**, various mixtures of gelator, solvent and compound **6** were heated to approximately 100 ºC until all the solid had dissolved and added to aliquots of simulant and allowed to cool to room temperature. DCP vapours were then added to the surface of the gel and allowed to diffuse into the gel structure leaving a colourless gel, as in Figure 4.

Gelator	Gelator Amount (mg)	Simulant	Simulant Amount (mL)	DMSO Amount (mL)	**6** Amount (mg)
**3**	12.00	DCP	0.10	1.00	0.00
**3**	12.00	DCP	0.10	1.00	10.00
**3**	12.00	DCP	0.10	1.00	40.00
**3**	12.00	DCP	0.10	1.00	160.00

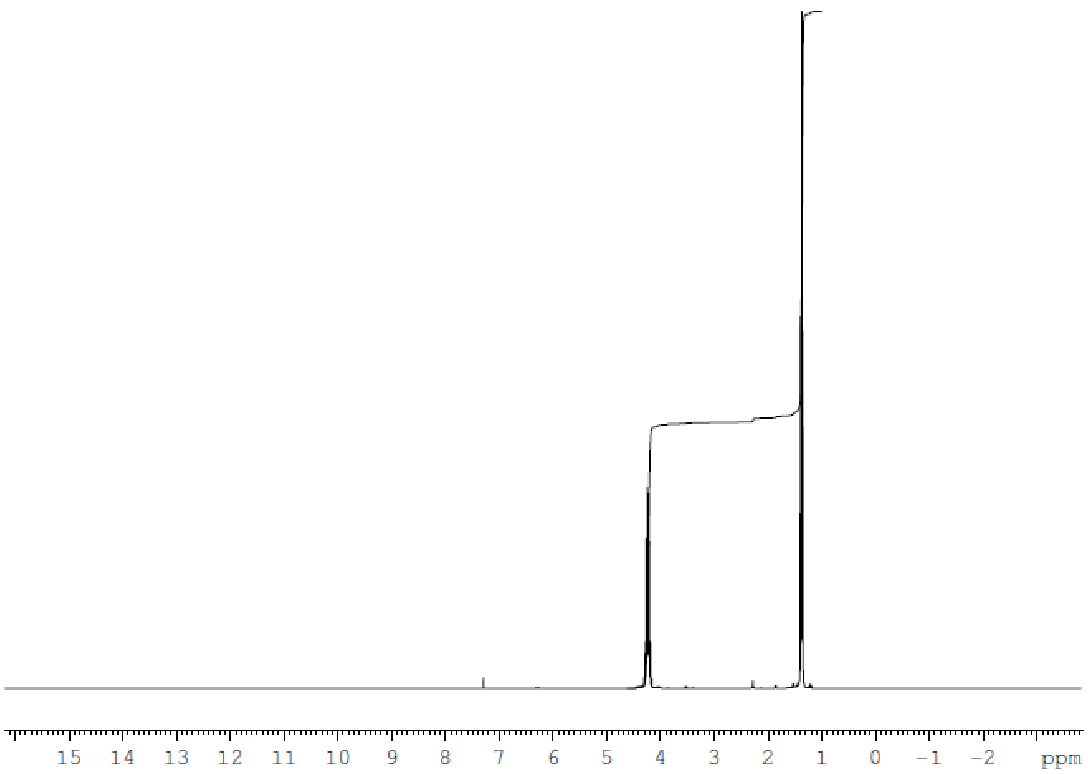

S18 ^{1}H NMR spectrum of DCP in $CDCl_3$.

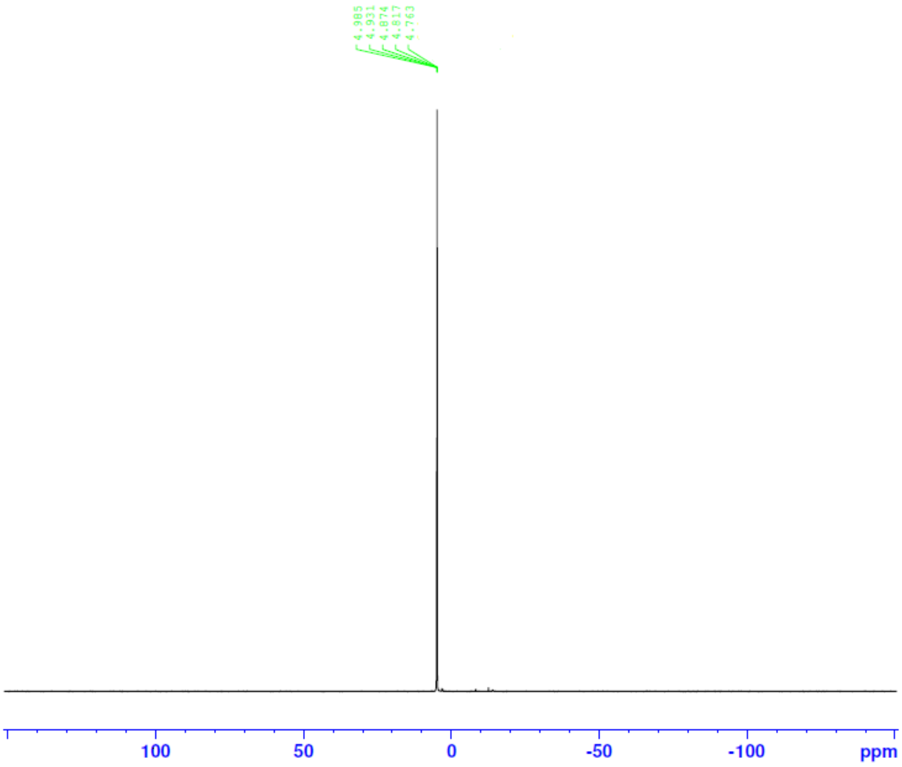

S19 ^{31}P NMR spectrum of DCP in $CDCl_3$.

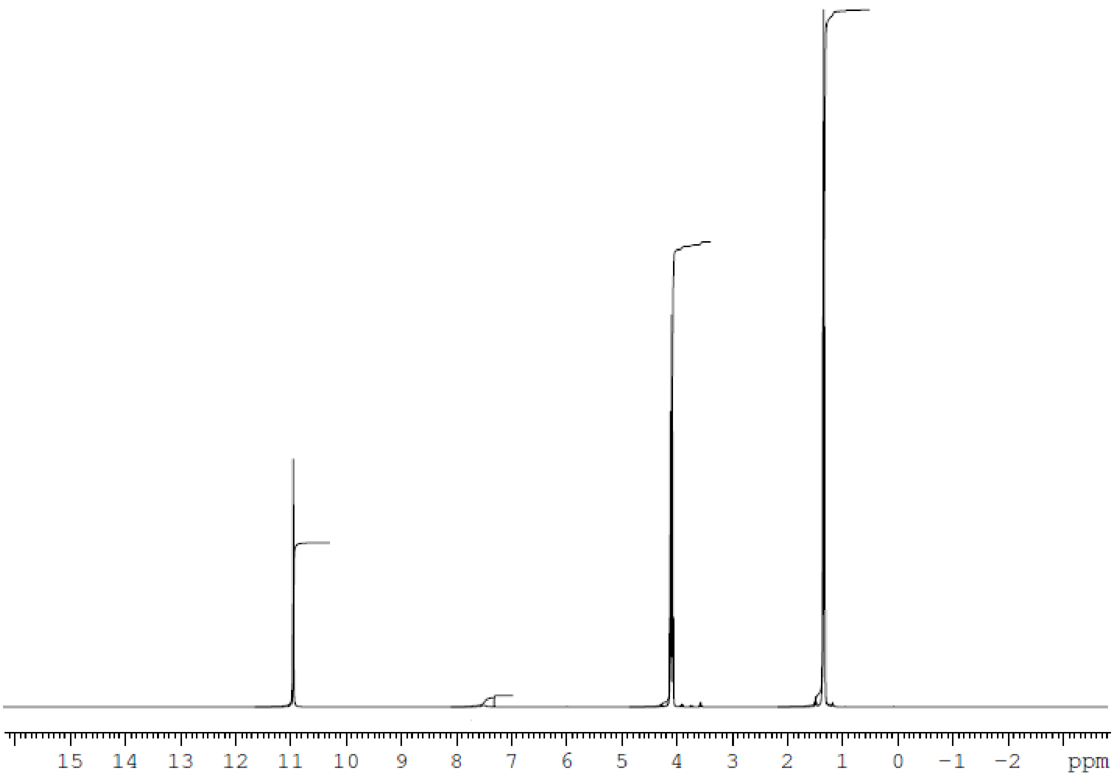

S20 ^{1}H NMR spectrum of DHP in $CDCl_3$.

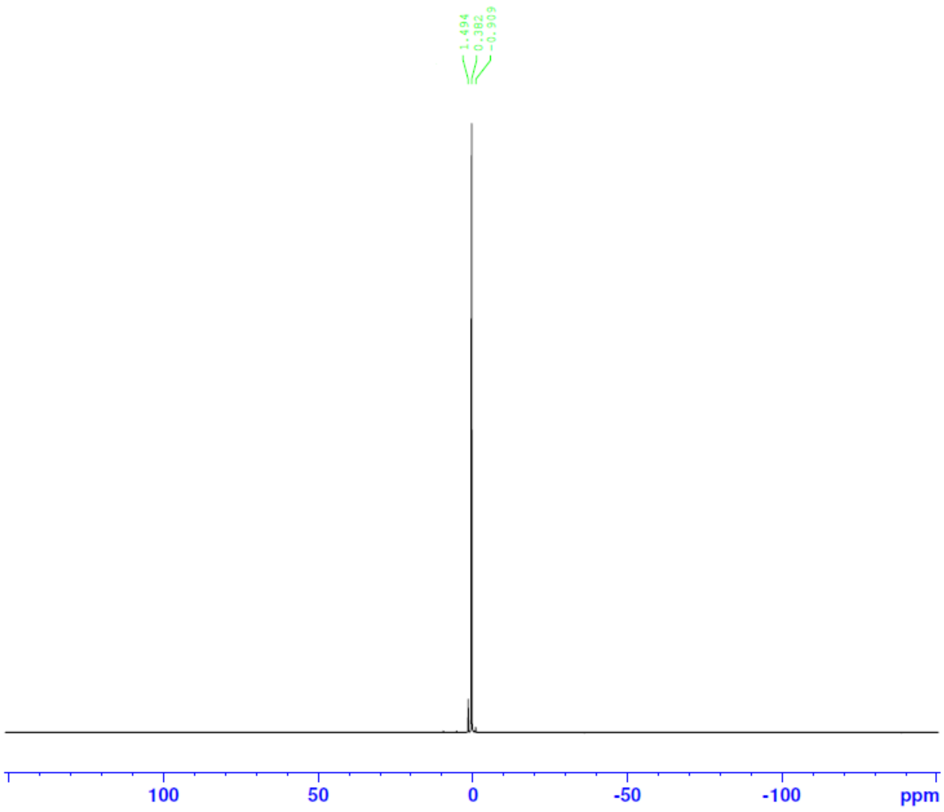

S21 ^{31}P NMR spectrum of DHP in $CDCl_3$.

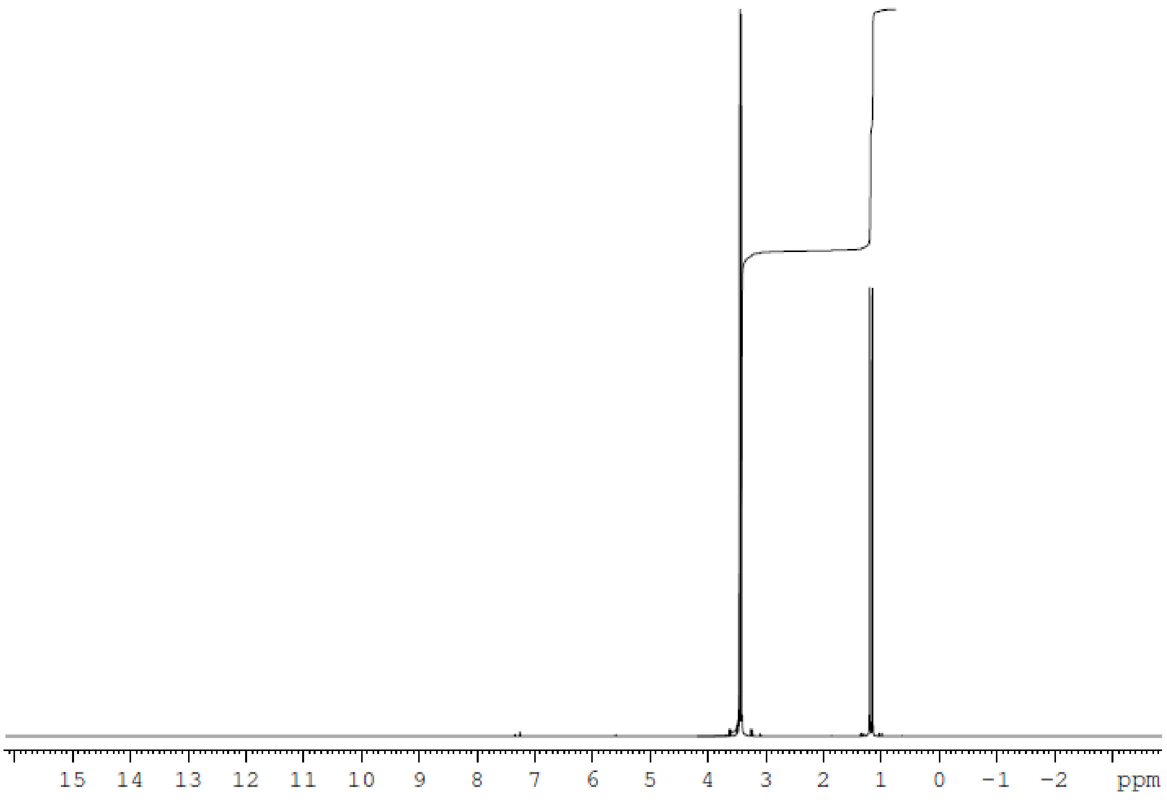

S22 ^{1}H NMR spectrum of DMMP in $CDCl_3$.

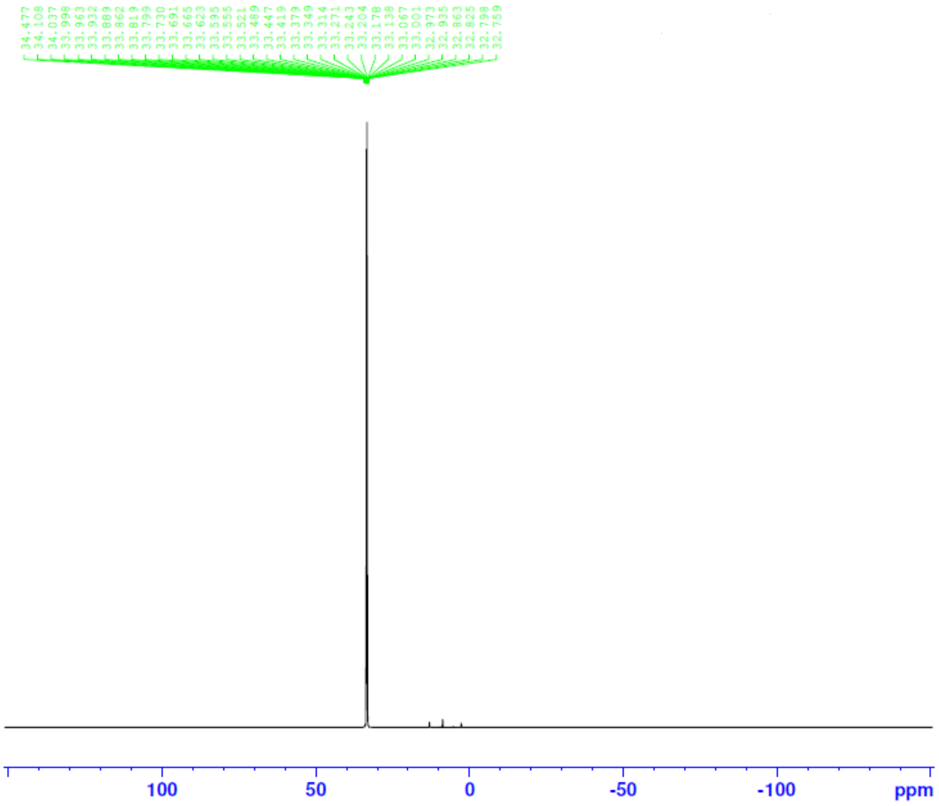

S23 ^{31}P NMR spectrum of DMMP in $CDCl_3$.

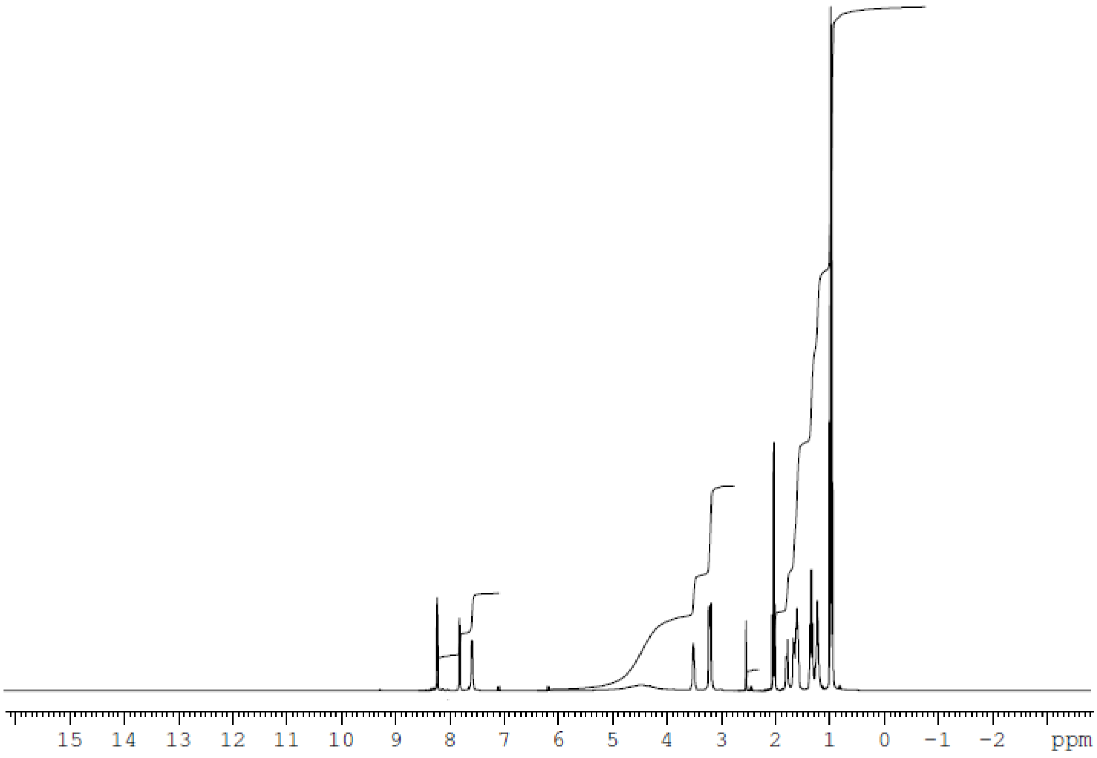

S24 ^{1}H NMR spectra in DMSO-d_6 (0.70 mL) of compound **5** (20 mg) and compound **6** (20 mg).

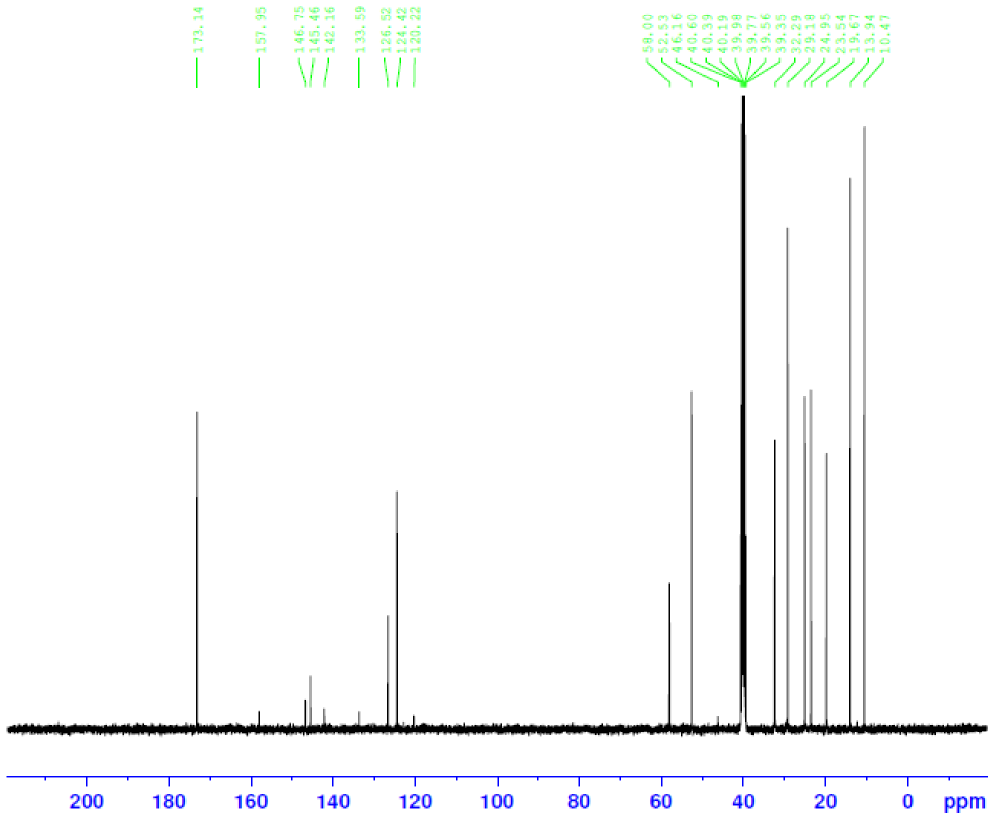

S25 ^{13}C NMR spectra in DMSO-d_6 (0.70 mL) of compound **5** (20 mg) and compound **6** (20 mg).

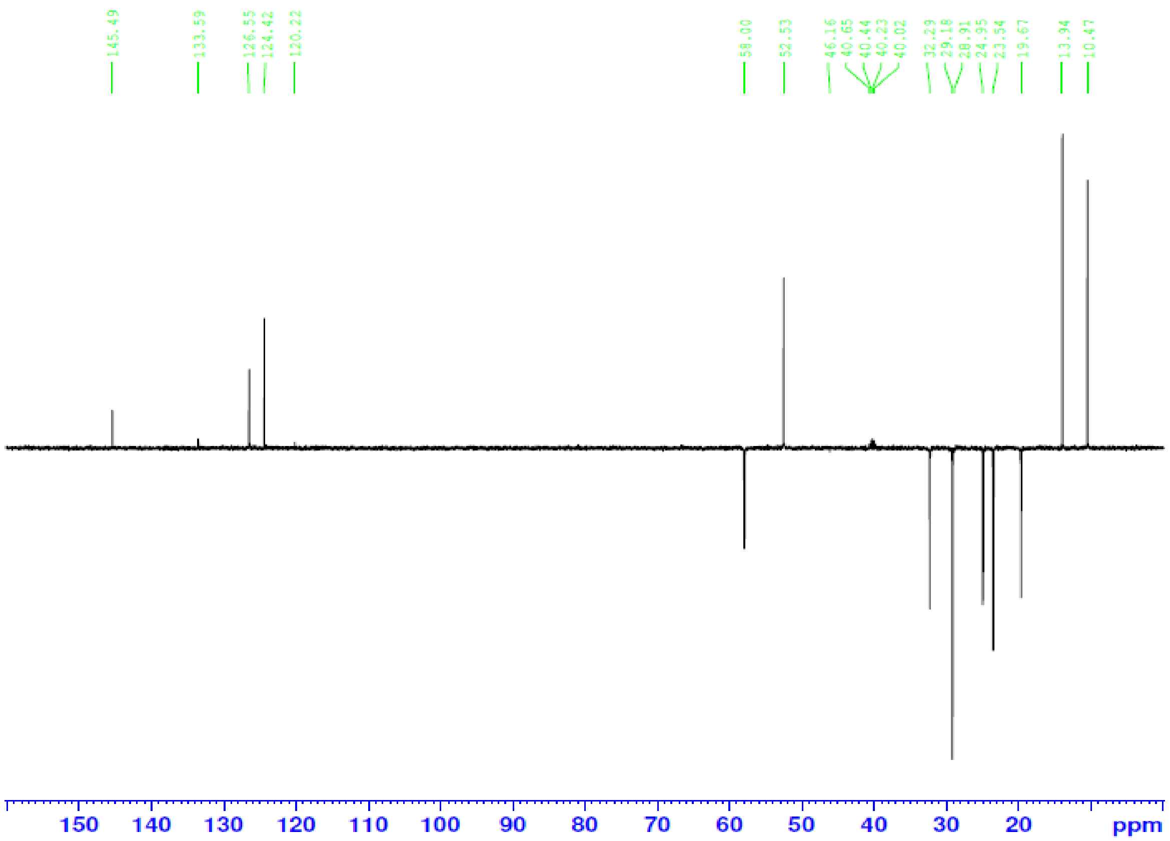

S26 DEPT135 NMR spectra in DMSO-d_6 (0.70 mL) of compound **5** (20 mg) and compound **6** (20 mg).

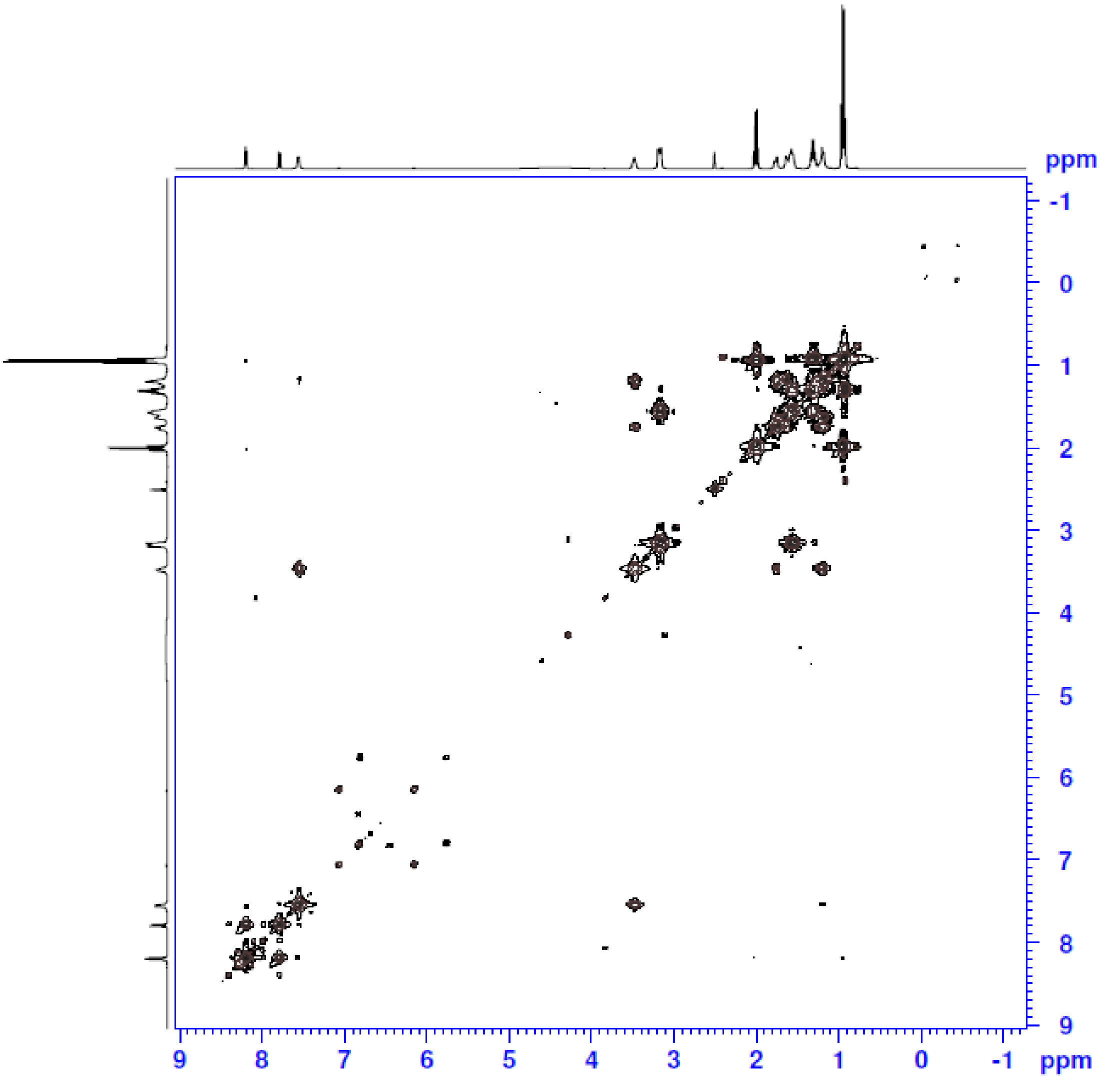

S27 ^{1}H COSY NMR spectra in DMSO-d_6 (0.70 mL) of compound **5** (20 mg) and compound **6** (20 mg).

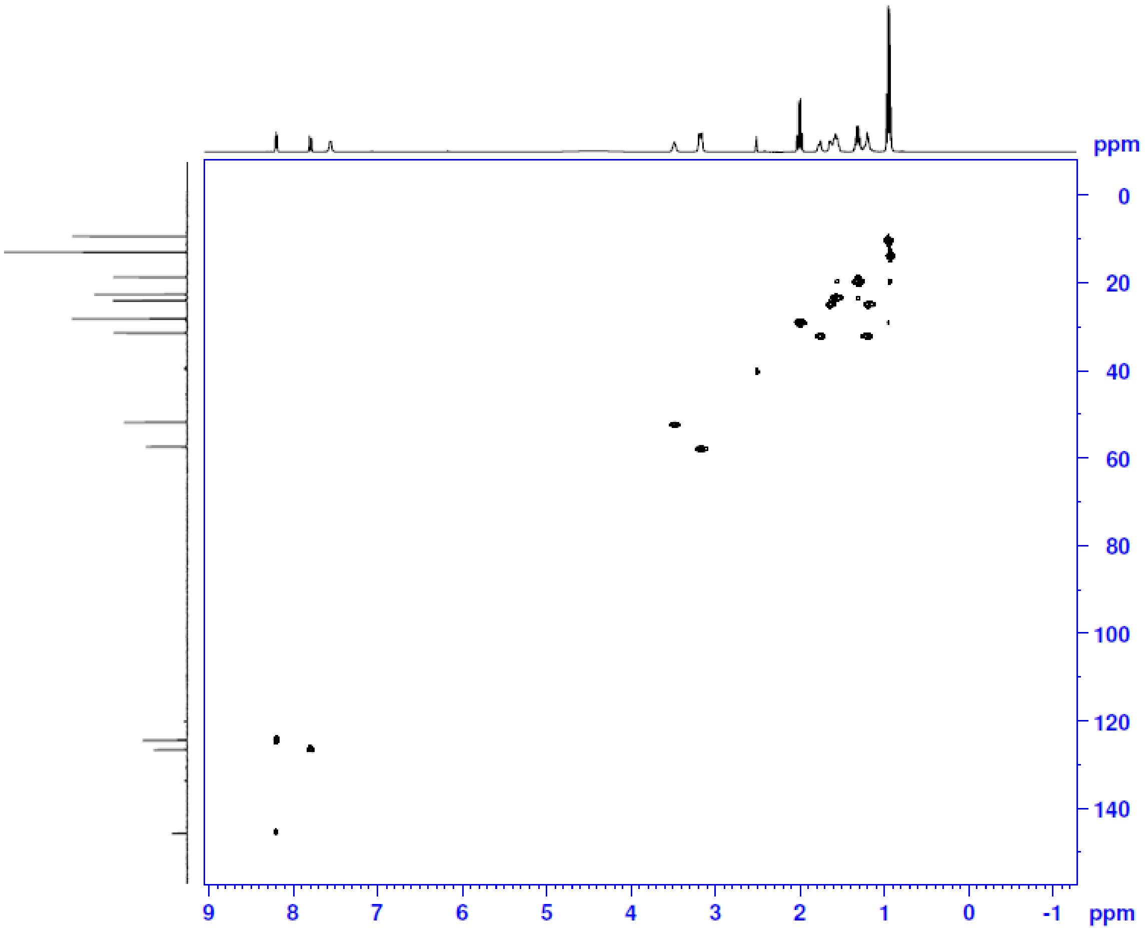

S28 HMQC NMR spectra in DMSO-d_6 (0.70 mL) of compound **5** (20 mg) and compound **6** (20 mg).

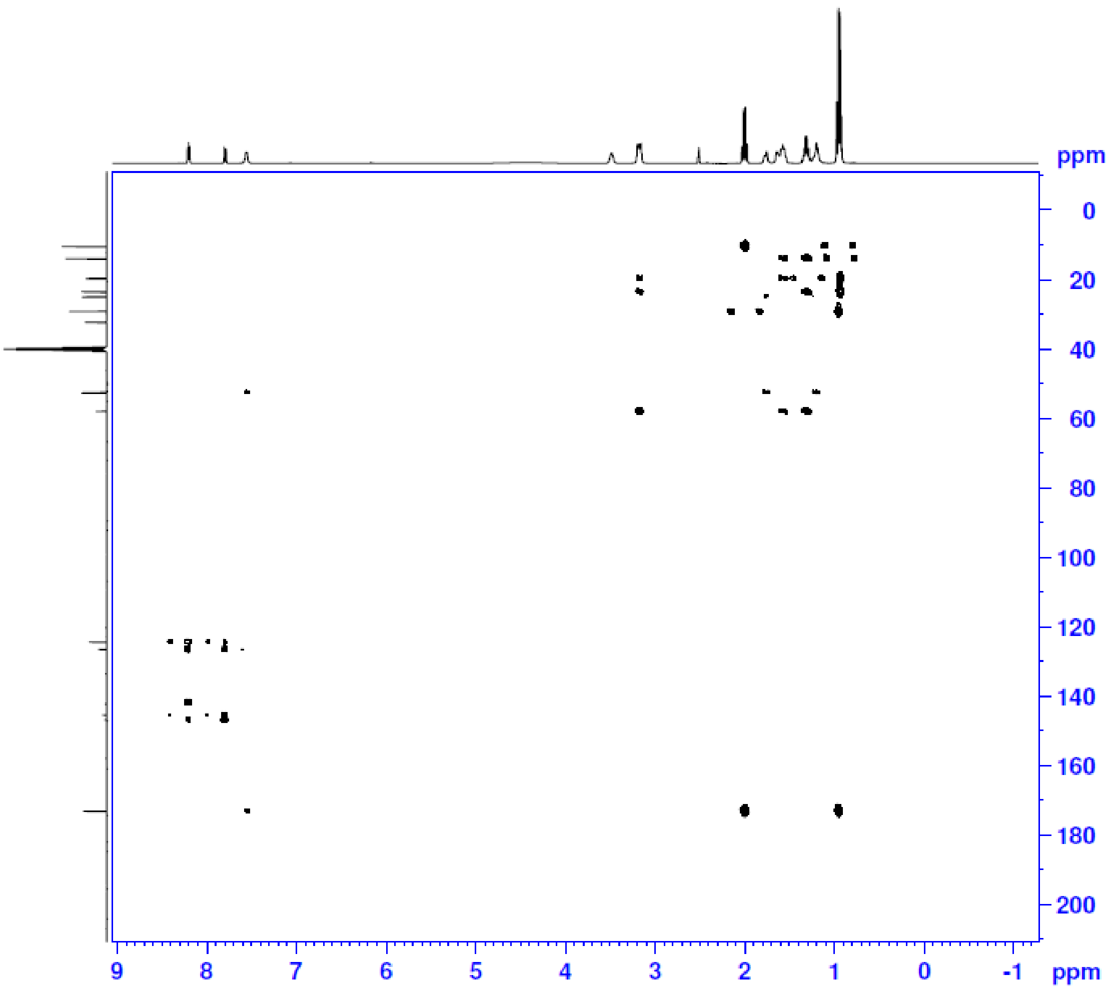

S29 HMBC NMR spectra in DMSO-d_6 (0.70 mL) of compound **5** (20 mg) and compound **6** (20 mg).

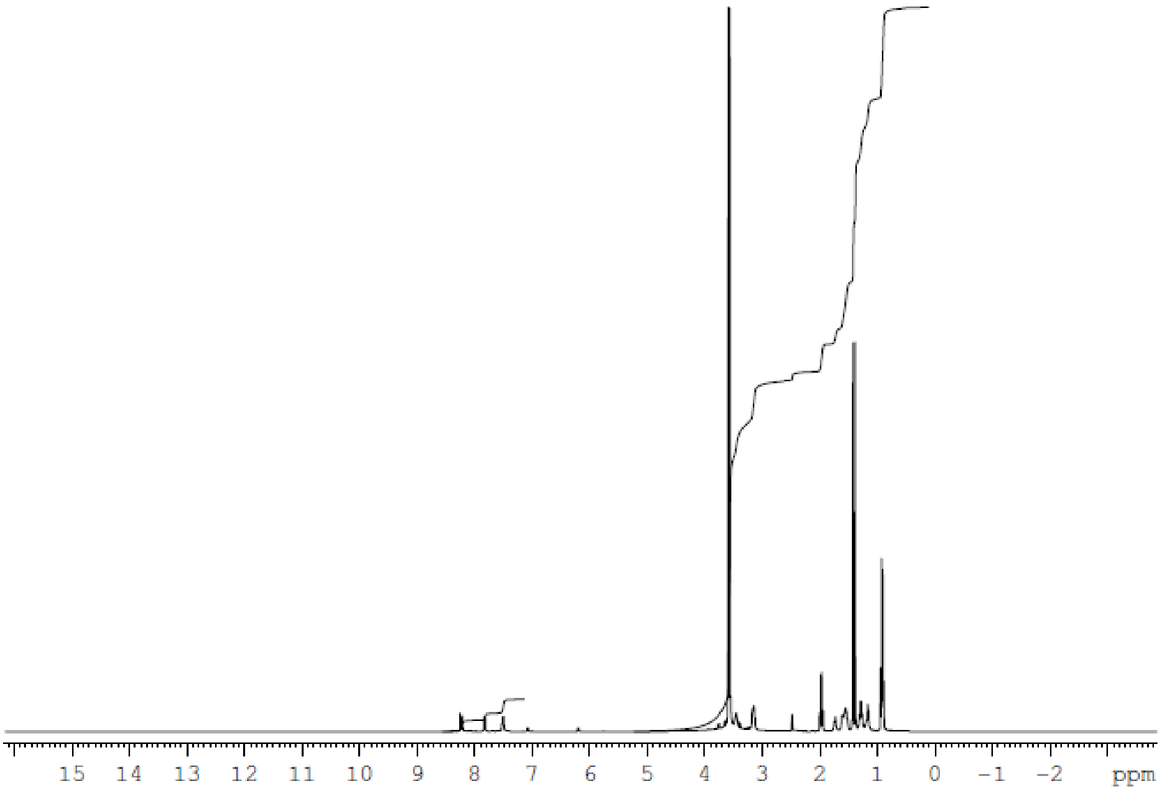

S30 ^{1}H NMR spectra in DMSO-d_6 (0.70 mL) of compound **5** (20 mg), DMMP (0.05 mL) and compound **6** (20 mg).

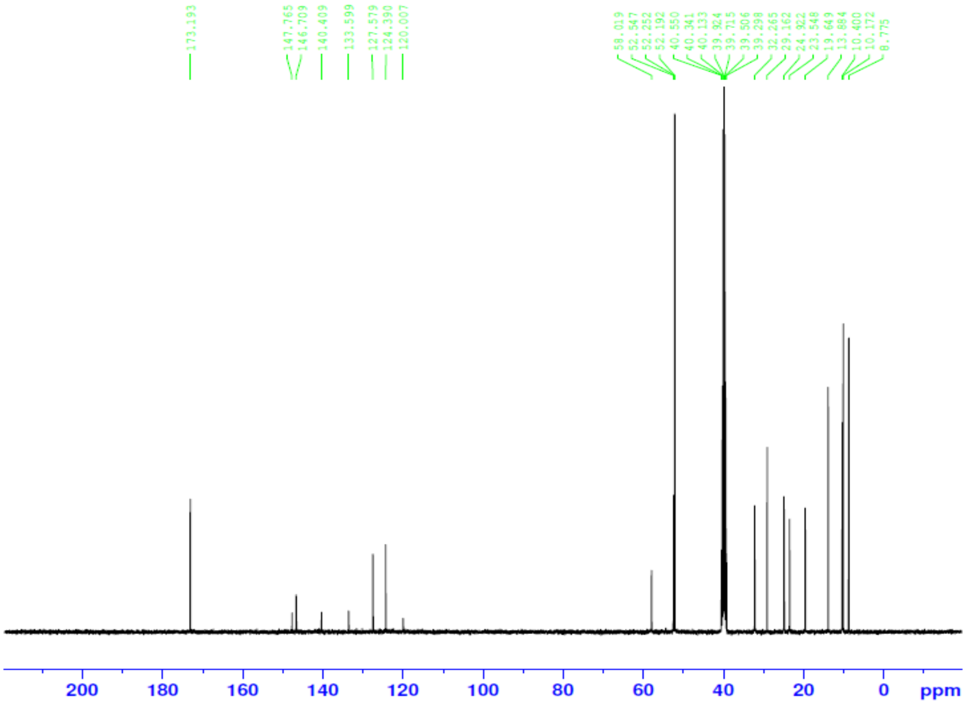

S31 ^{13}C NMR spectra in DMSO-d_6 (0.70 mL) of compound **5** (20 mg), DMMP (0.05 mL) and compound **6** (20 mg).

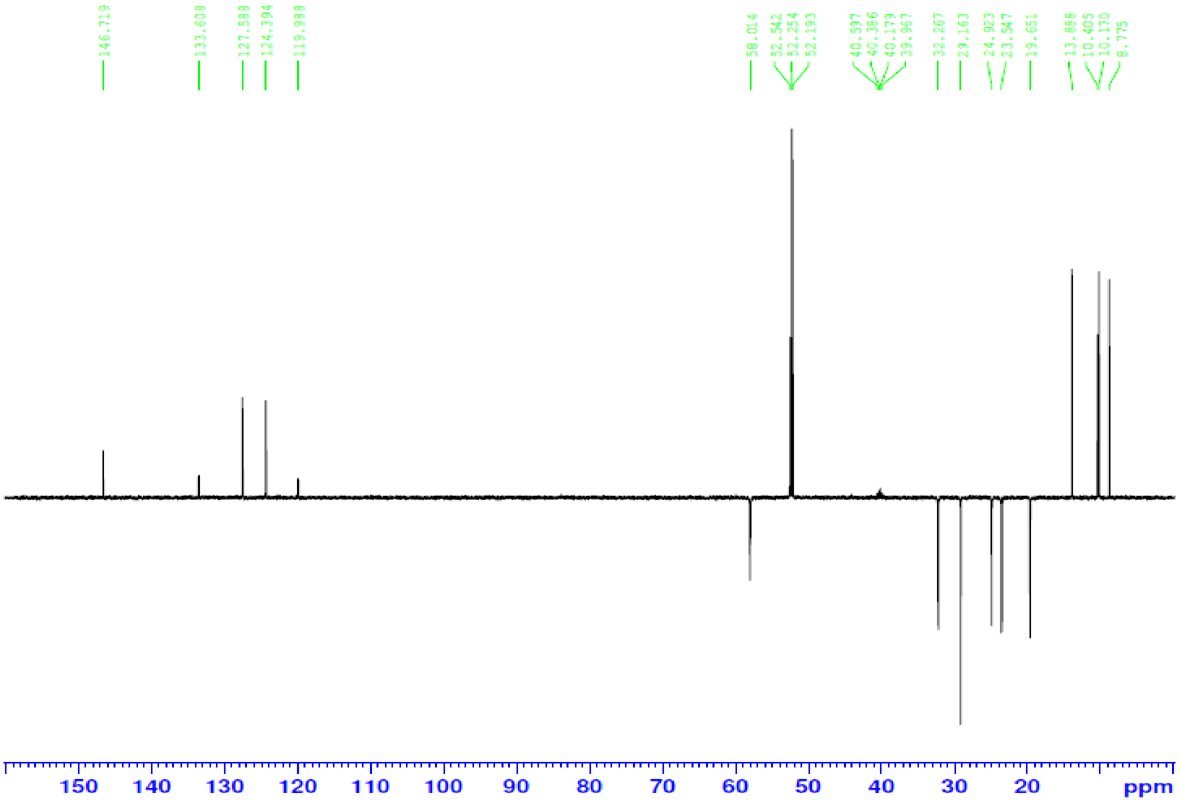

S32 DEPT135 NMR spectra in DMSO-d_6 (0.70 mL) of compound **5** (20 mg), DMMP (0.05 mL) and compound **6** (20 mg).

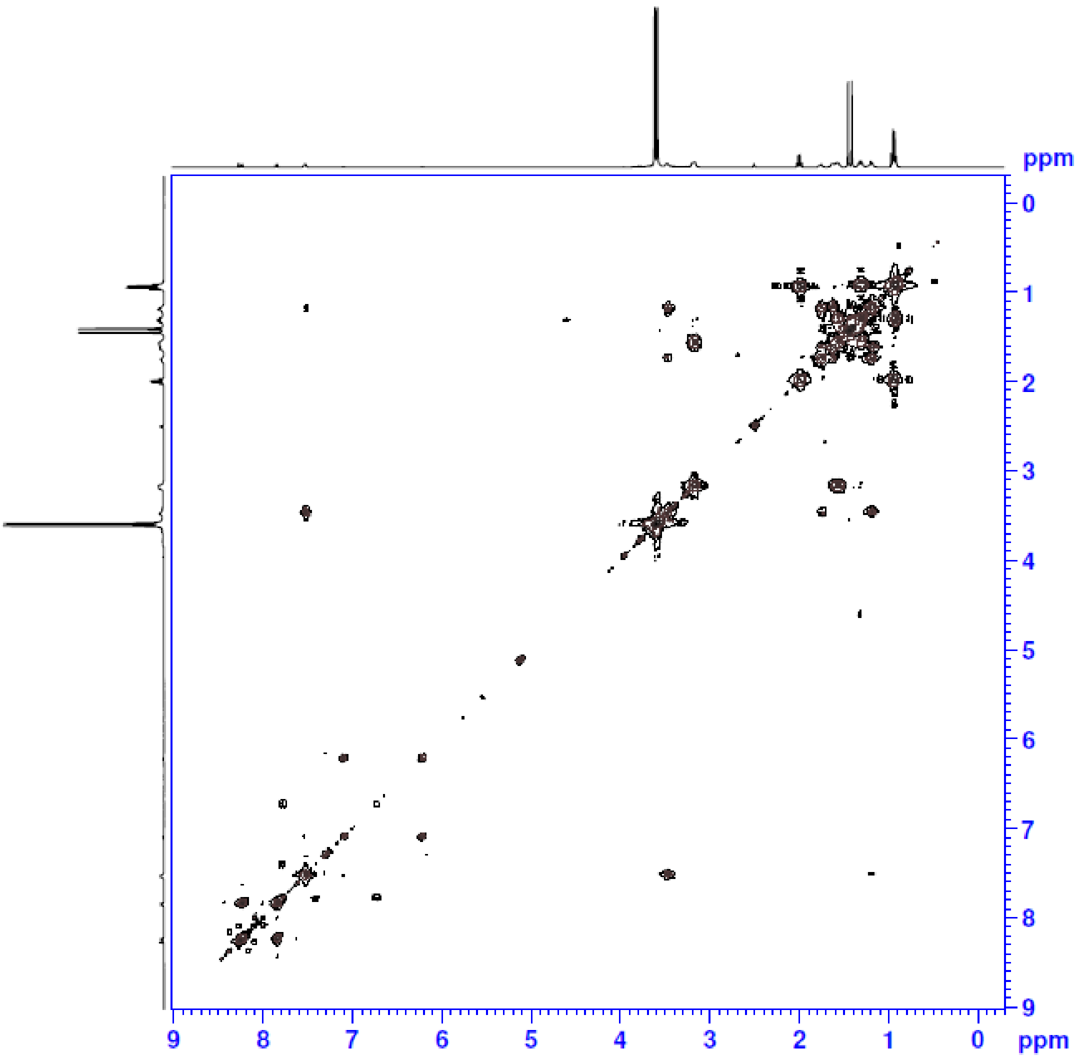

S33 ^{1}H COSY NMR spectra in DMSO-d_6 (0.70 mL) of compound **5** (20 mg), DMMP (0.05 mL) and compound **6** (20 mg).

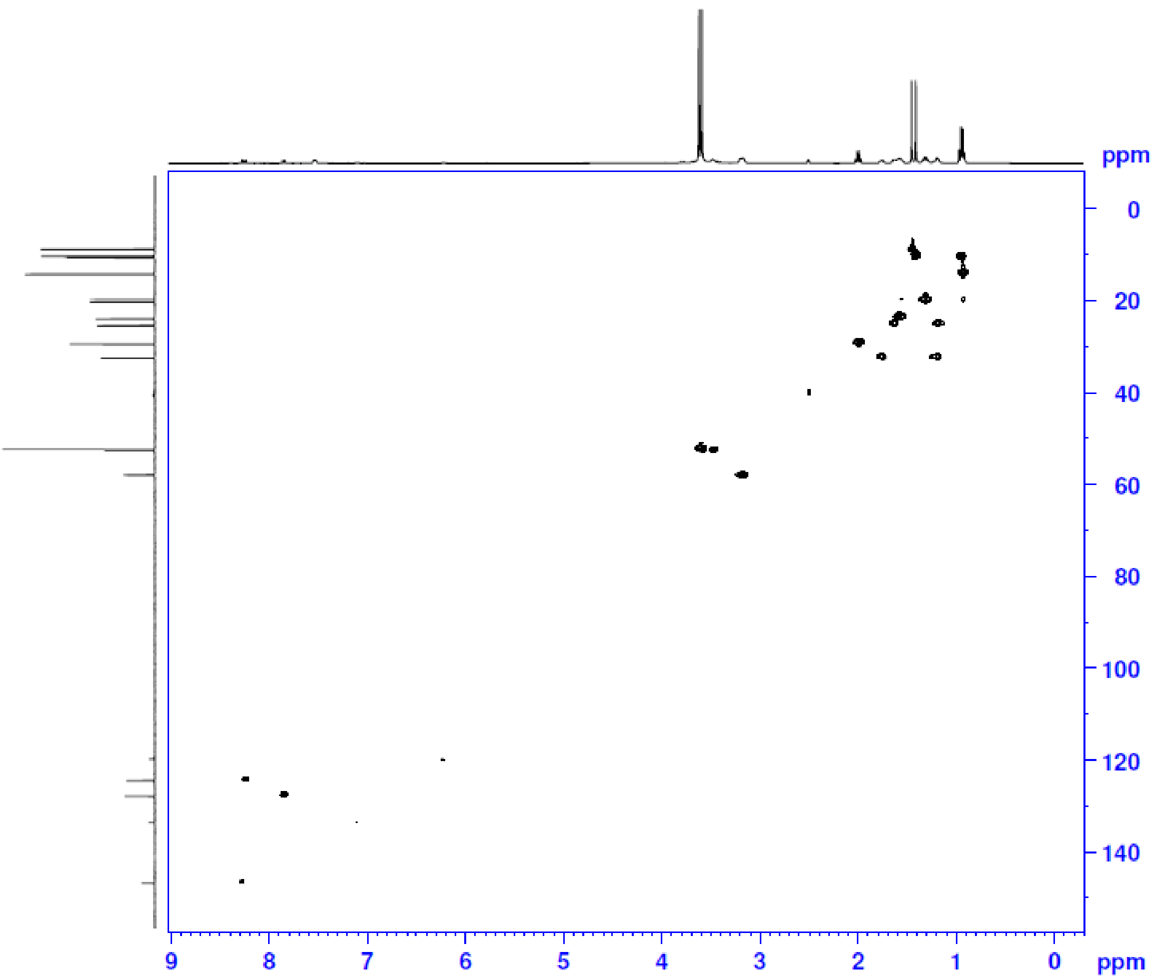

S34 HMQC NMR spectra in DMSO-d_6 (0.70 mL) of compound **5** (20 mg), DMMP (0.05 mL) and compound **6** (20 mg).

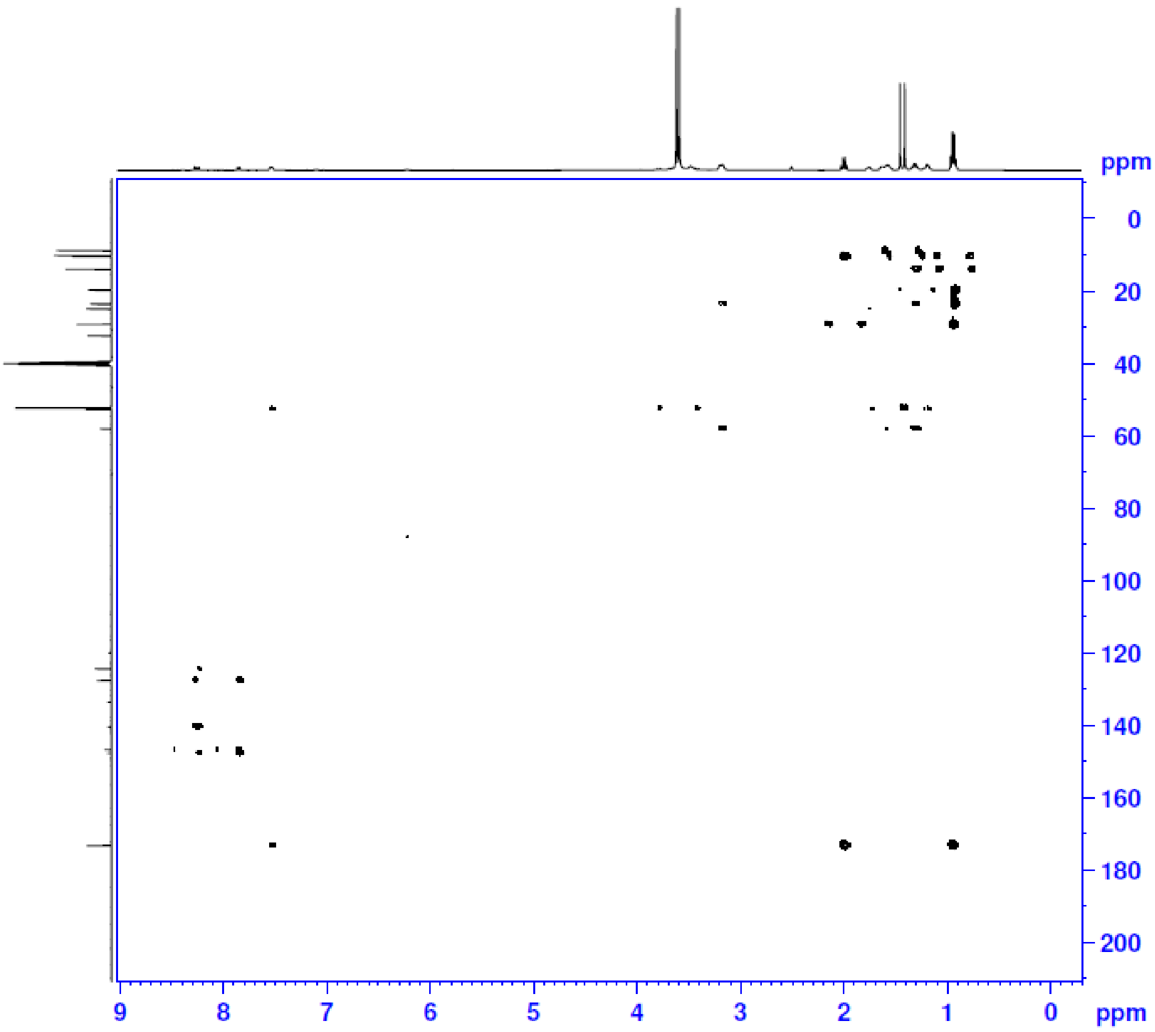

S35 HMBC NMR spectra in DMSO-d_6 (0.70 mL) of compound **5** (20 mg), DMMP (0.05 mL) and compound **6** (20 mg).

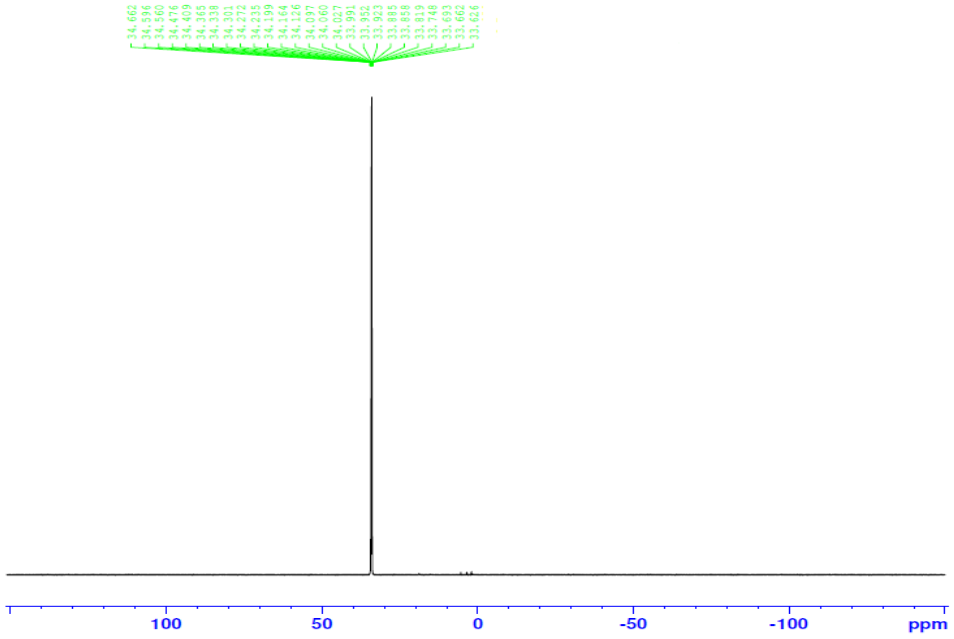

S36 ^{31}P NMR spectra in DMSO-d_6 (0.70 mL) of compound **5** (20 mg), DMMP (0.05 mL) and compound **6** (20 mg).

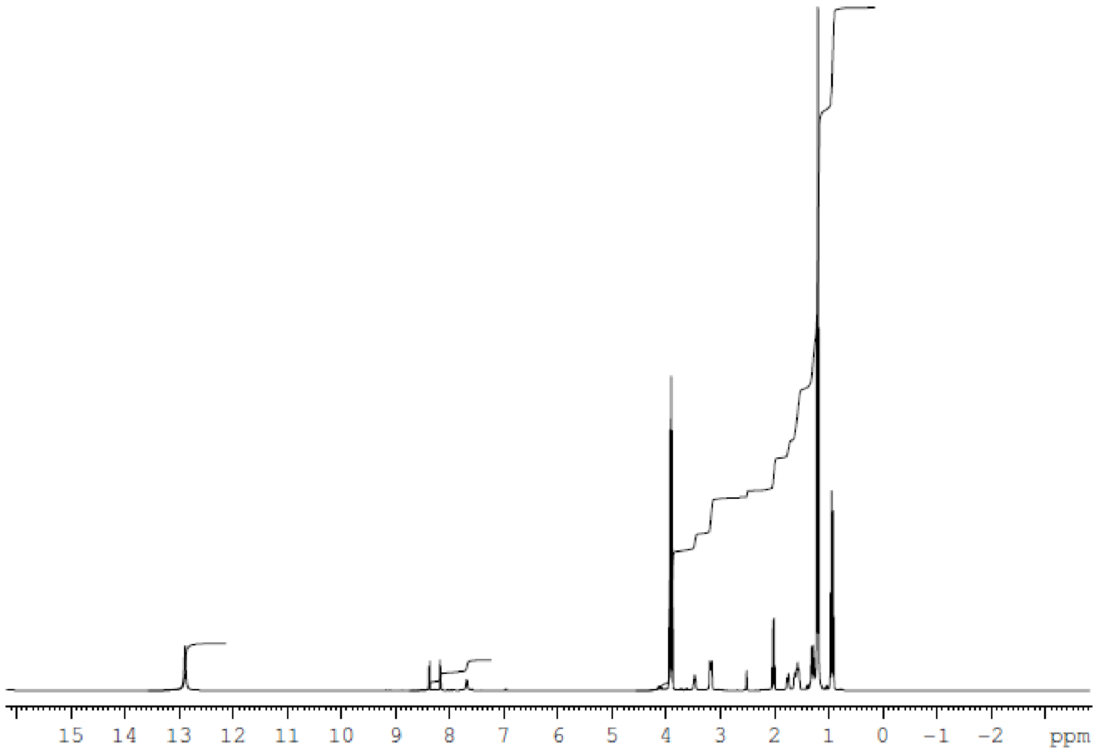

S37 ^{1}H NMR spectra in DMSO-d_6 (0.70 mL) of compound **5** (20 mg), DCP (0.05 mL) and compound **6** (20 mg).

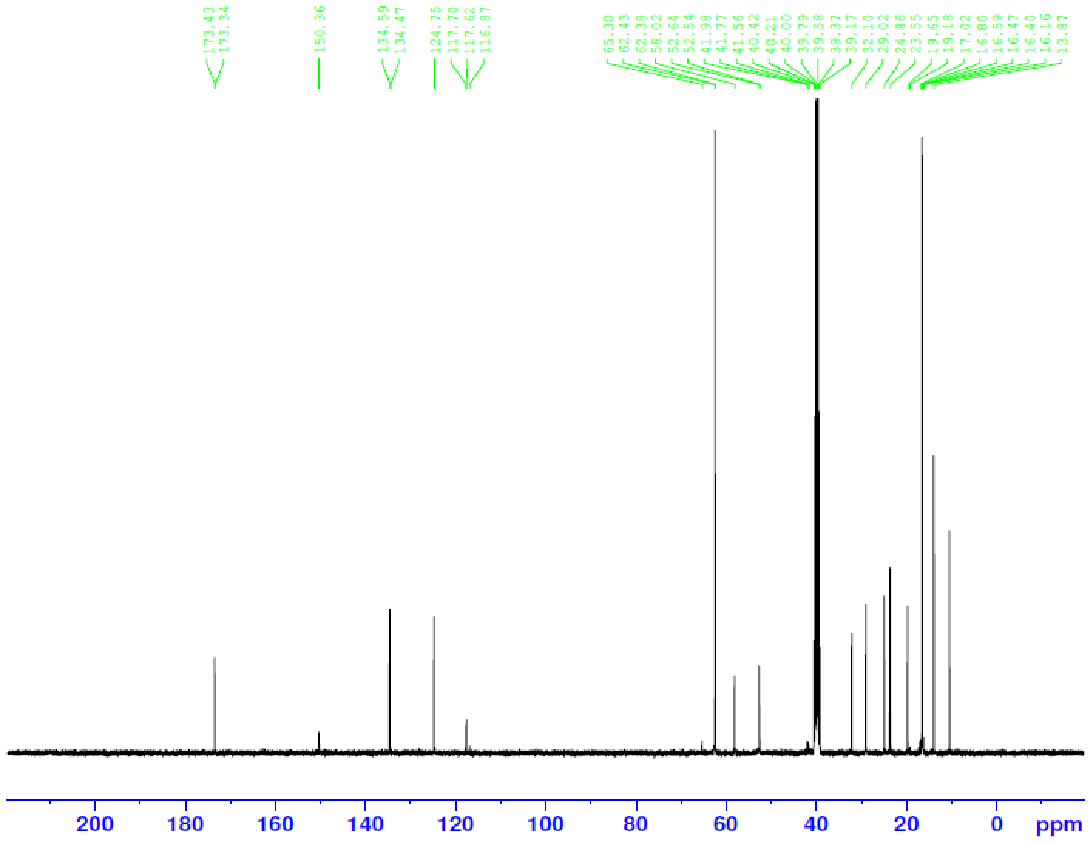

S38 ^{13}C NMR spectra in DMSO-d_6 (0.70 mL) of compound **5** (20 mg), DCP (0.05 mL) and compound **6** (20 mg).

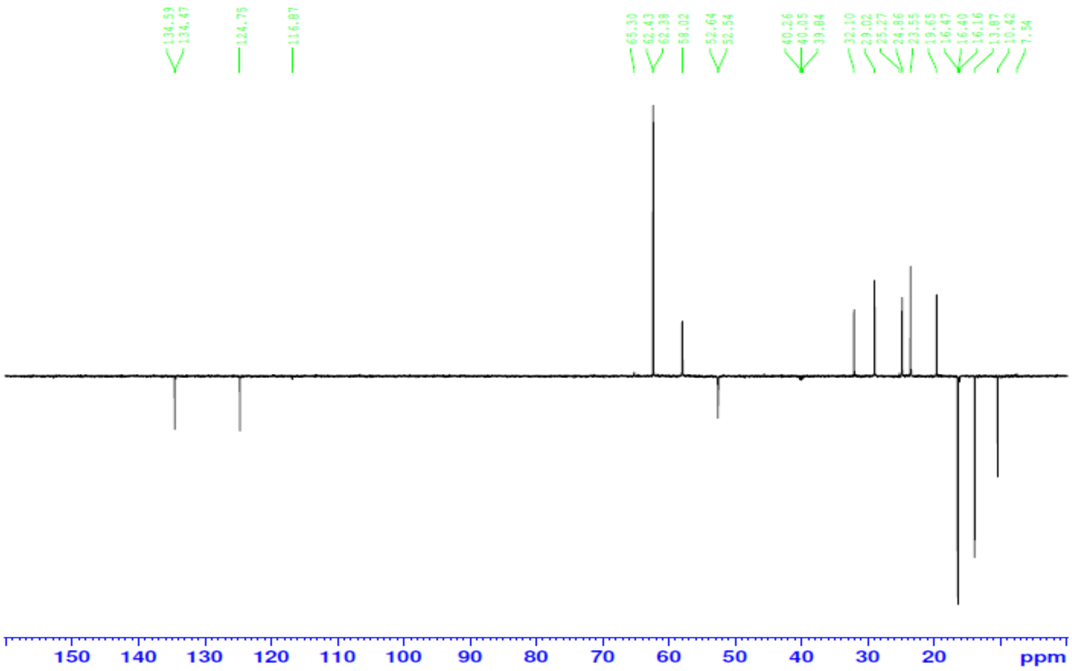

S39 DEPT135 NMR spectra in DMSO-d_6 (0.70 mL) of compound **5** (20 mg), DCP (0.05 mL) and compound **6** (20 mg).

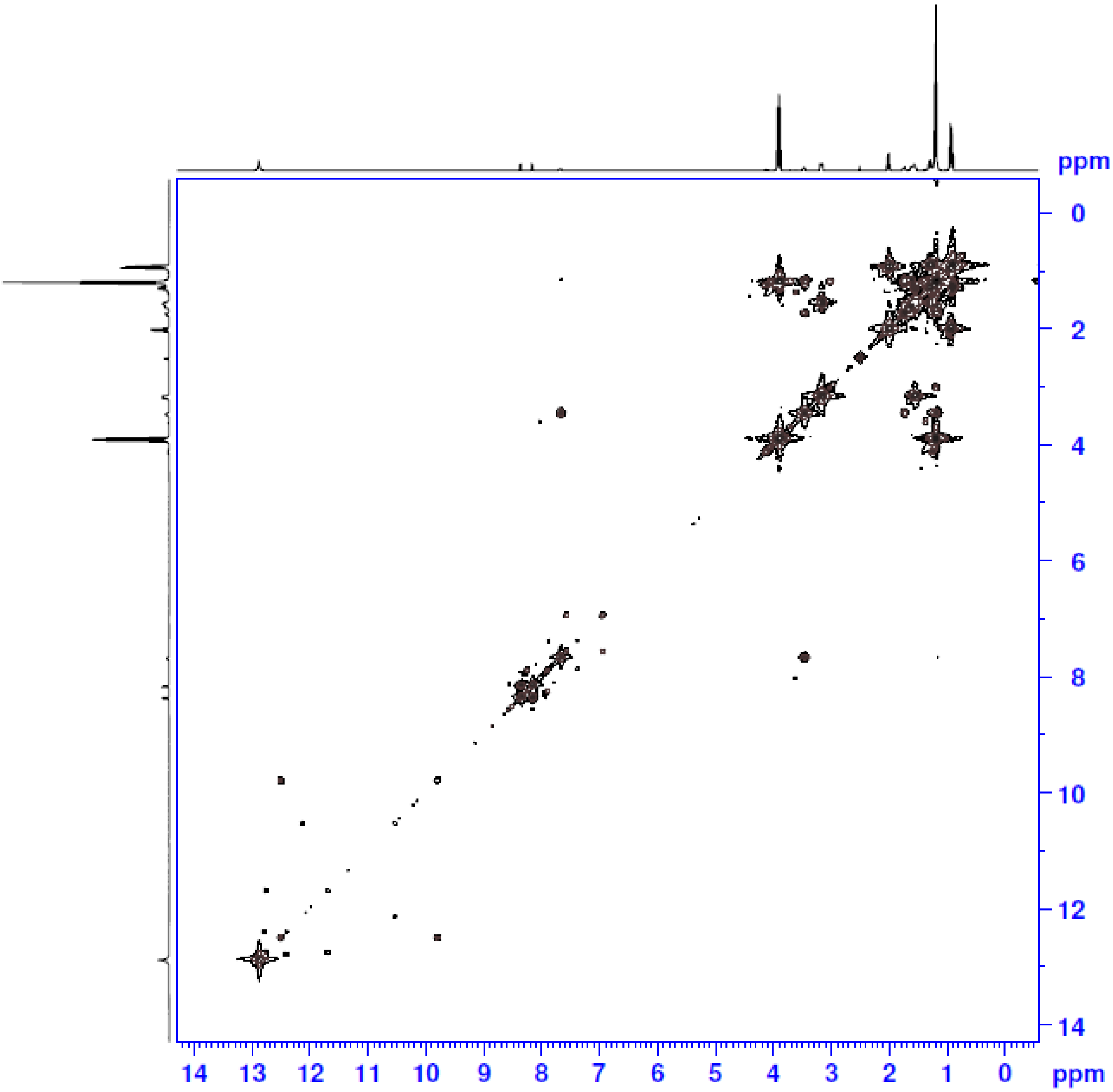

S40 ^{1}H COSY NMR spectra in DMSO-d_6 (0.70 mL) of compound **5** (20 mg), DCP (0.05 mL) and compound **6** (20 mg).

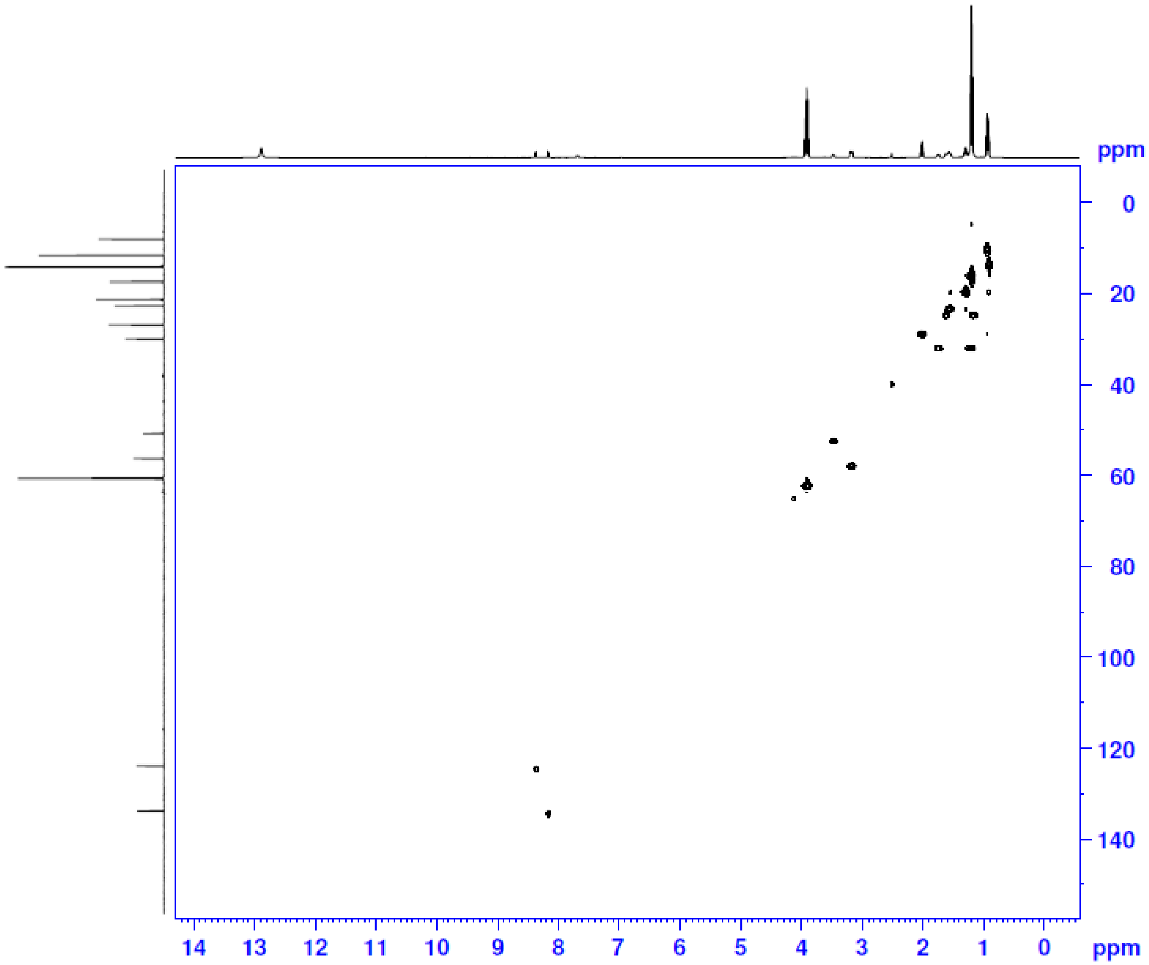

S41 HMQC NMR spectra in DMSO-d_6 (0.70 mL) of compound **5** (20 mg), DCP (0.05 mL) and compound **6** (20 mg).

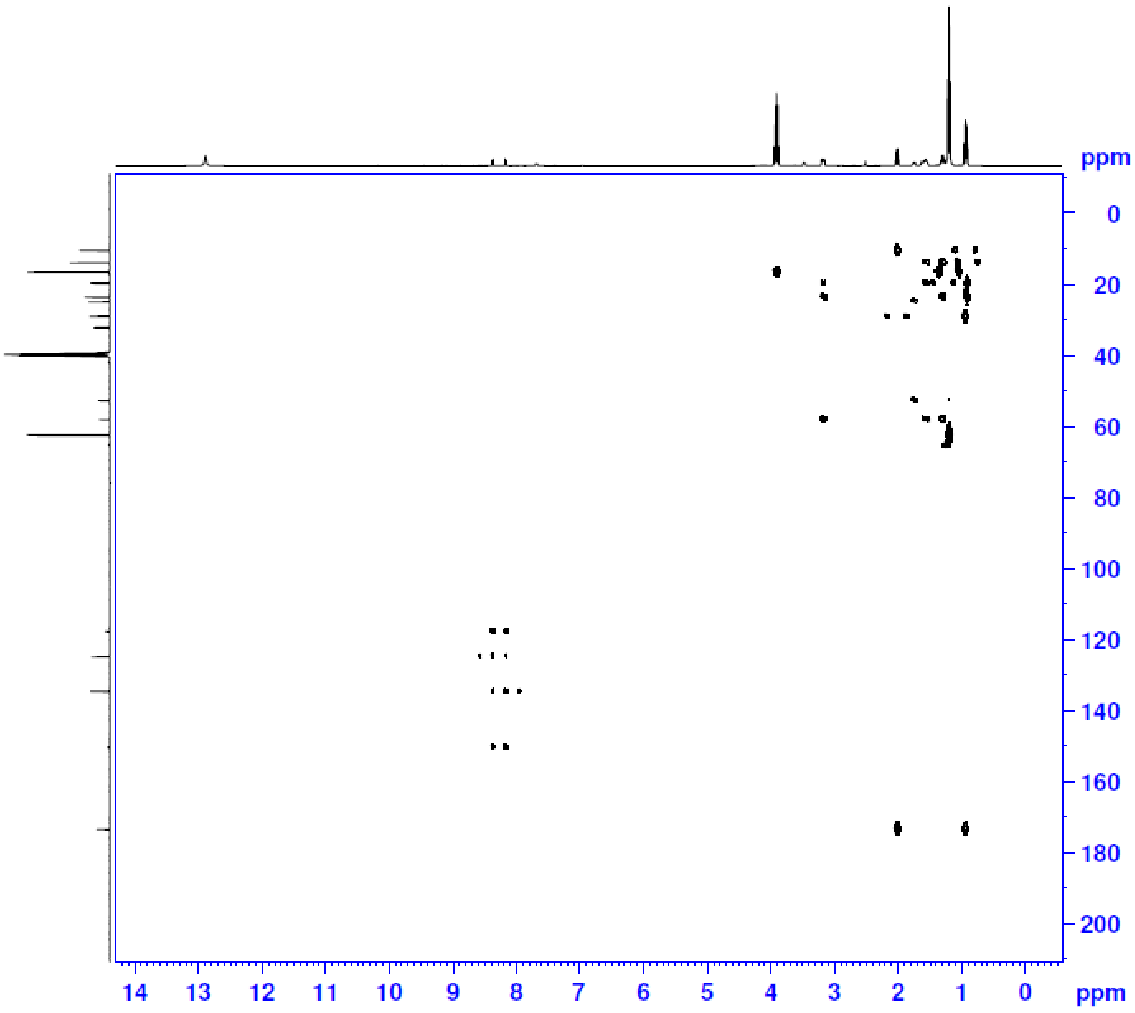

S42 HMBC NMR spectra in DMSO-d_6 (0.70 mL) of compound **5** (20 mg), DCP (0.05 mL) and compound **6** (20 mg).

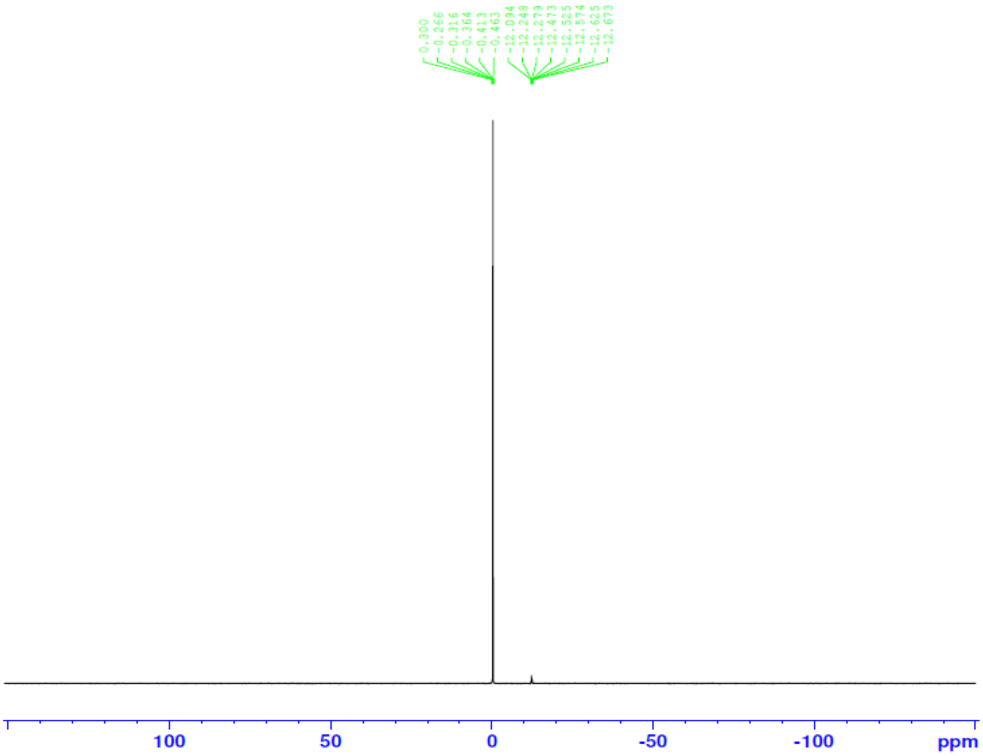

S43 ^{31}P NMR spectra in DMSO-d_6 (0.70 mL) of compound **5** (20 mg), DCP (0.05 mL) and compound **6** (20 mg).

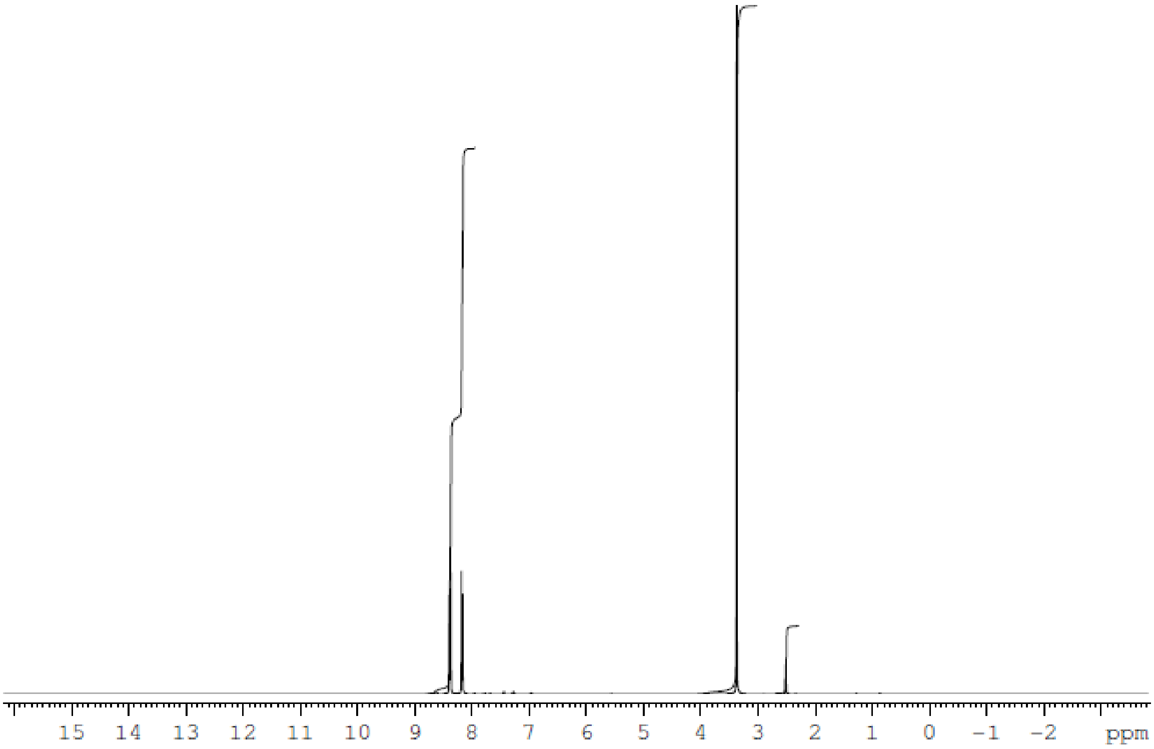

S44 ^{1}H NMR spectra in DMSO-d_6 (0.70 mL) of compound **7** (20 mg).

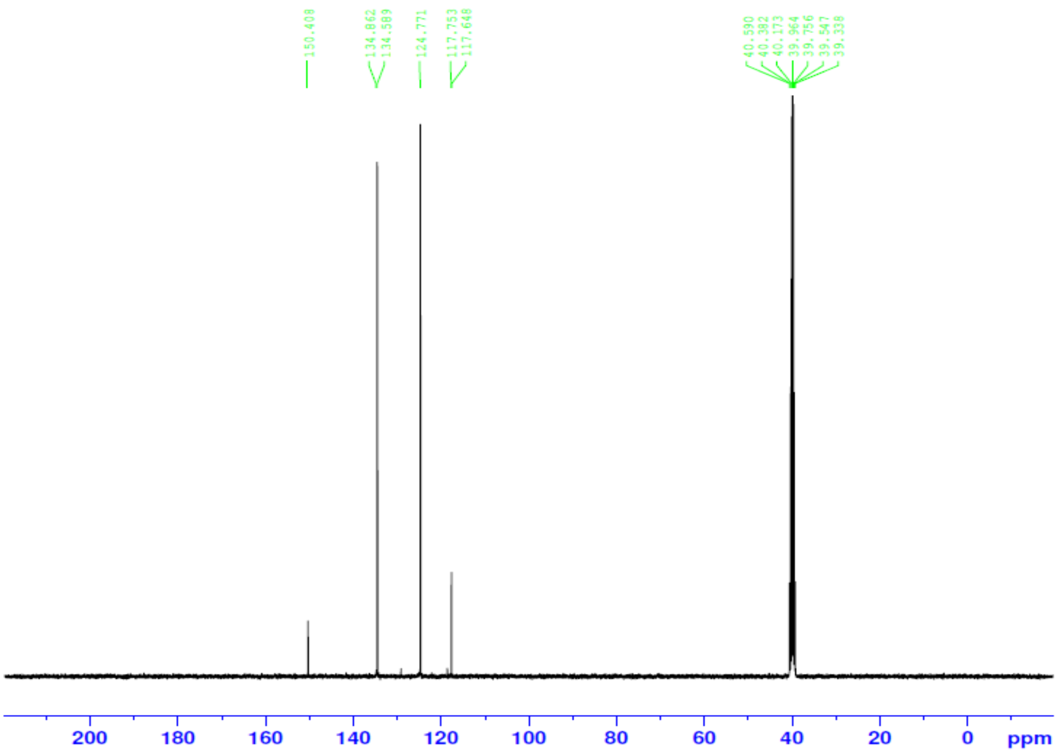

S45 ^{31}C NMR spectra in DMSO-d_6 (0.70 mL) of compound **7** (20 mg).

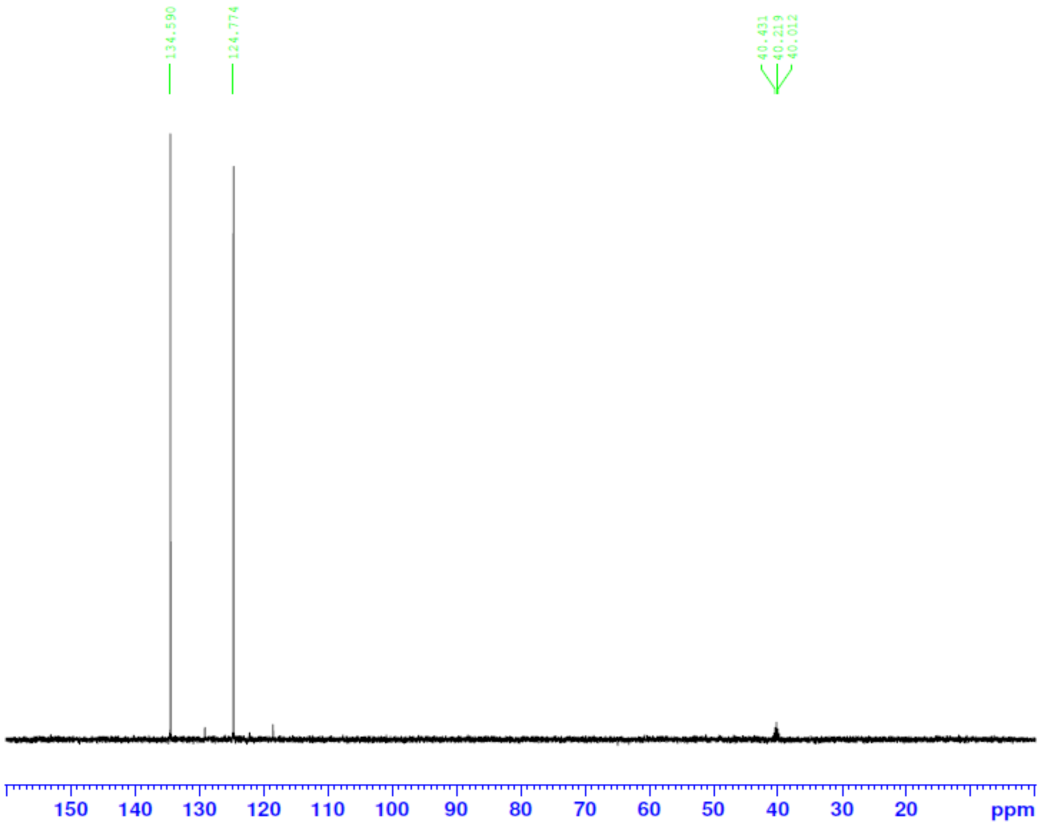

S46 DEPT135 NMR spectra in DMSO-d_6 (0.70 mL) of compound **7** (20 mg).

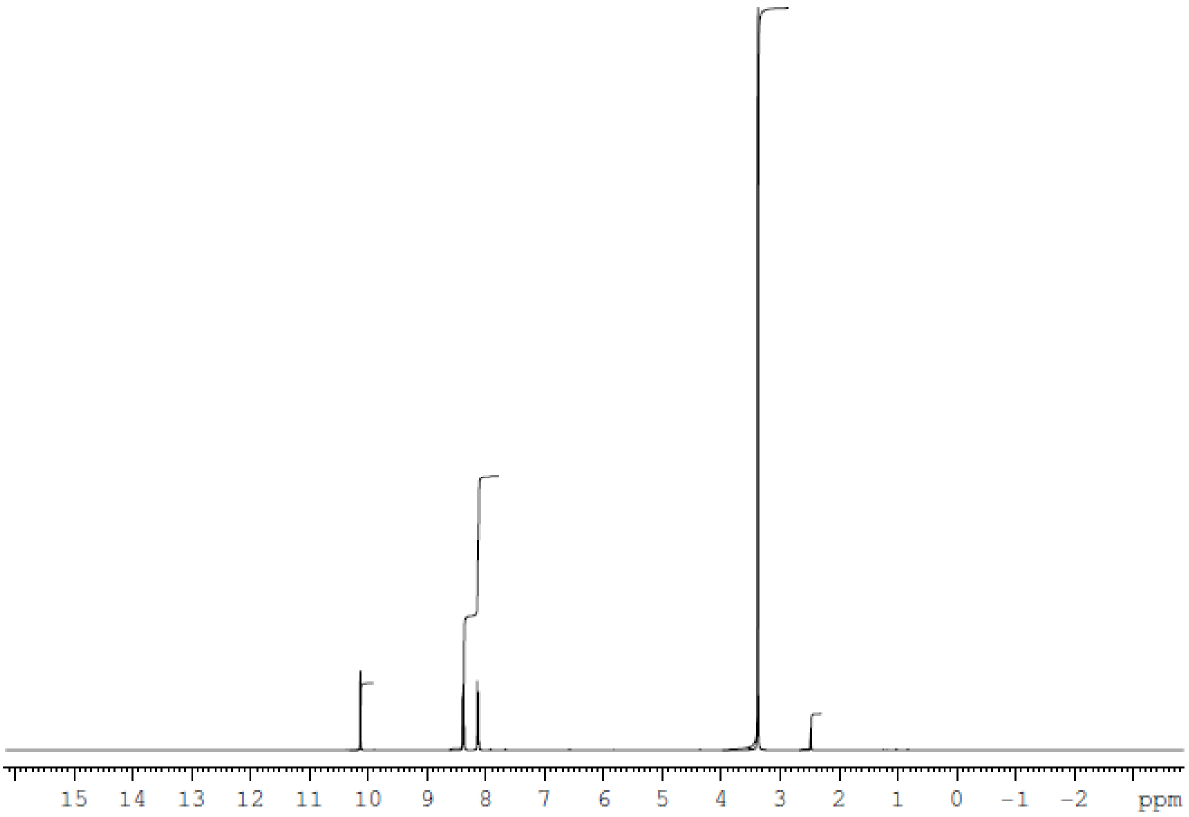

S47 ^{1}H NMR spectra in DMSO-d_6 (0.70 mL) of 4-nitrobenzaldehyde (20 mg).

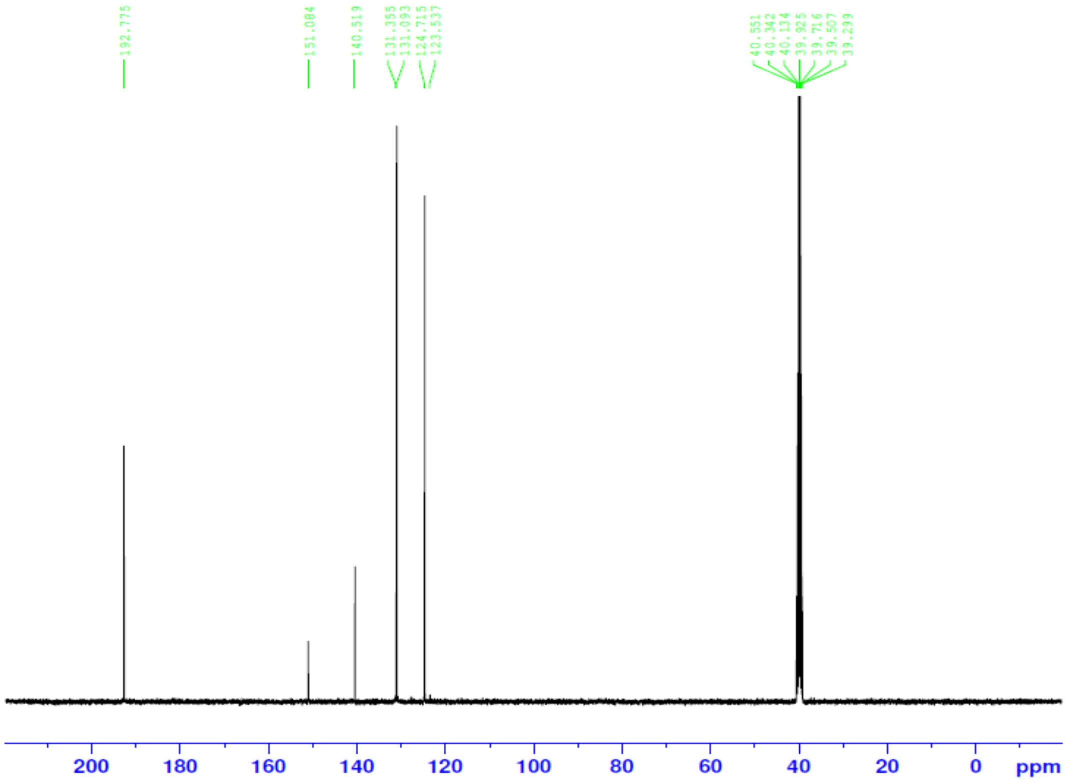

S48 ^{13}C NMR spectra in DMSO-d_6 (0.70 mL) of 4-nitrobenzaldehyde (20 mg).

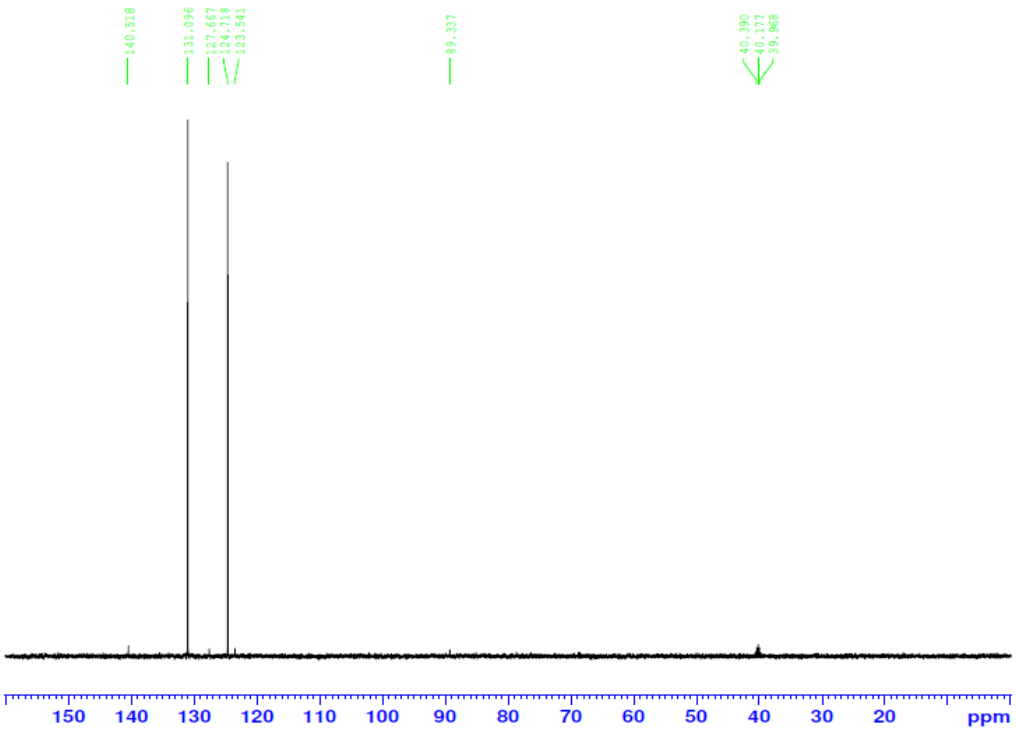

S49 DEPT135 NMR spectra in DMSO-d_6 (0.70 mL) of 4-nitrobenzaldehyde (20 mg).

Organogel NMR experiments

An organogel was synthesised using deuterated DMSO (0.5 mL), compound **6** (80 mg) and compound **3** (11.5 mg). This sample was placed into an NMR tube (5 mm OD) and allowed to cool to room temperature. DCP (0.1 mL) was added to the surface of the NMR tube which was then sealed. The surface of the gel was approximately 4 mm above the top of the NMR aperture, which was approximately 23 mm in length. The reaction with compound **6** was then observed as the DCP front moved into the aperture observable by the NMR. The signals corresponding to the reacted and unreacted compound **6** were then integrated and used to calculate the proportion of unreacted compound **6** visible in the NMR aperture. The results and line of best fitting are shown in S50. This experimental method is limited by the resolution of the NMR experiment due to the different phases present in the sample.

Two further identical samples were synthesised as previously described. To one of the samples, while it was still a warm sol before setting, was added DCP (0.1 mL). The sol containing DCP was then transferred to an NMR tube and allowed to set. NMR experiments were then performed on both the organogel with and without the presence of DCP to check the reactions within the gel. ^{1}H NMR spectra of gel sample without DCP added gave signals in the region 9-6 ppm correspond to the oximate CH's. In this case they appear in two different environments, due to the different states within the gel and/or the two different isomers that this molecule can adopt. There was no evidence of this however in the solid state so is most likely an effect due to gel formation.

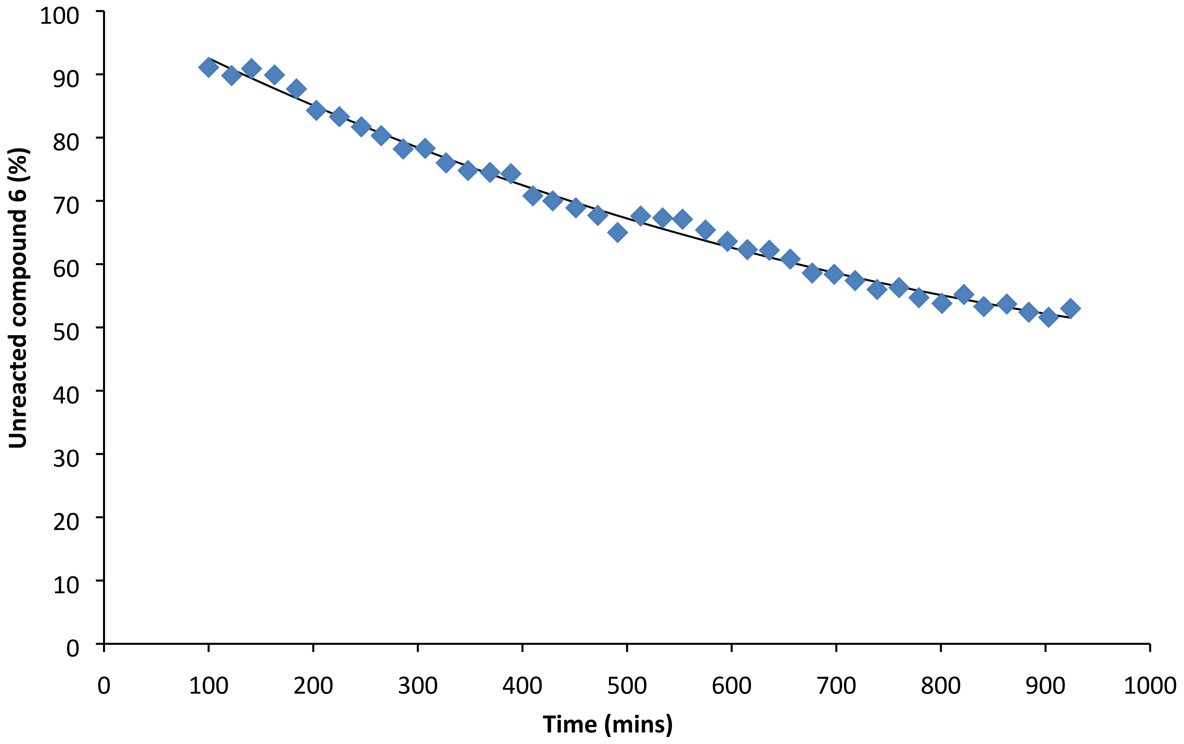

S50 Effects of DCP (0.1 mL) addition to the surface of an organogel (DMSO-d_6 (0.5 mL), compound **6** (80 mg) and compound **3** (11.5 mg)) with respect to time.

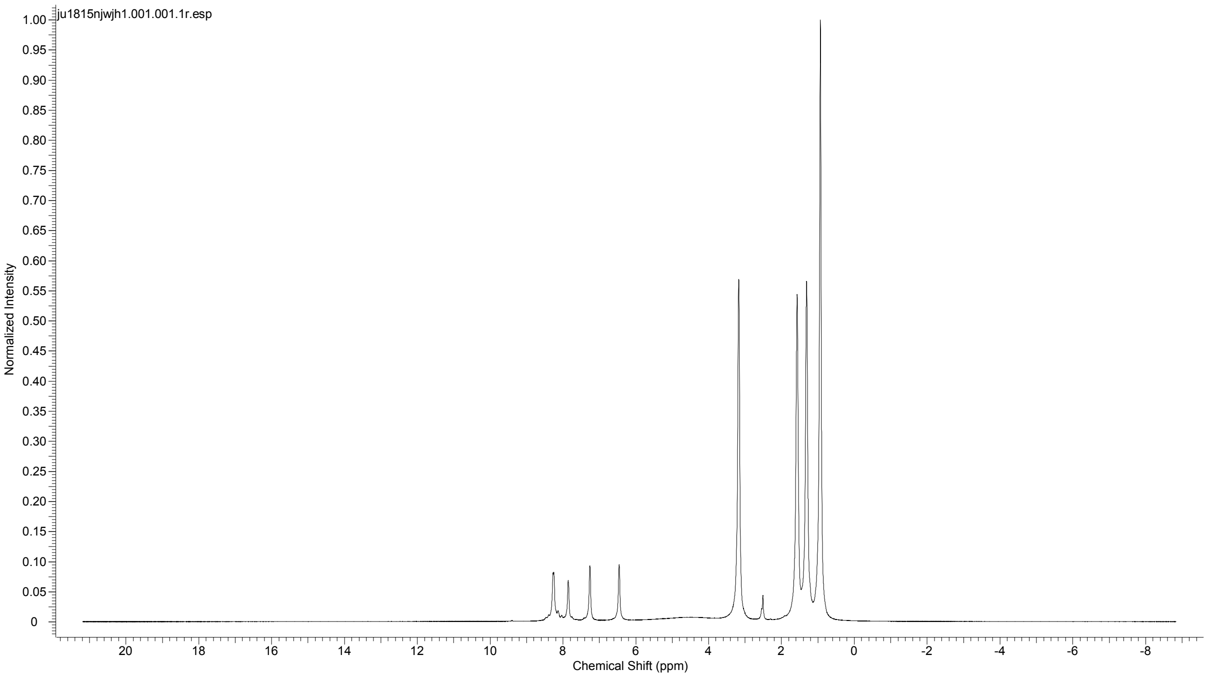

S51 ^{1}H NMR spectra of an organogel (DMSO-d_6 (0.5 mL), compound **6** (80 mg) and compound **3** (11.5 mg)). The integrals of the peaks in the region 9-6 ppm correspond to the oximate CH's. In this case they appear in two different environments, due to the different states within the gel and/or the two different isomers that this molecule can adopt. There was no evidence of this however in the solid state so is most likely an effect due to gel formation.

ju1815njwjh1.002.001.2rr.esp

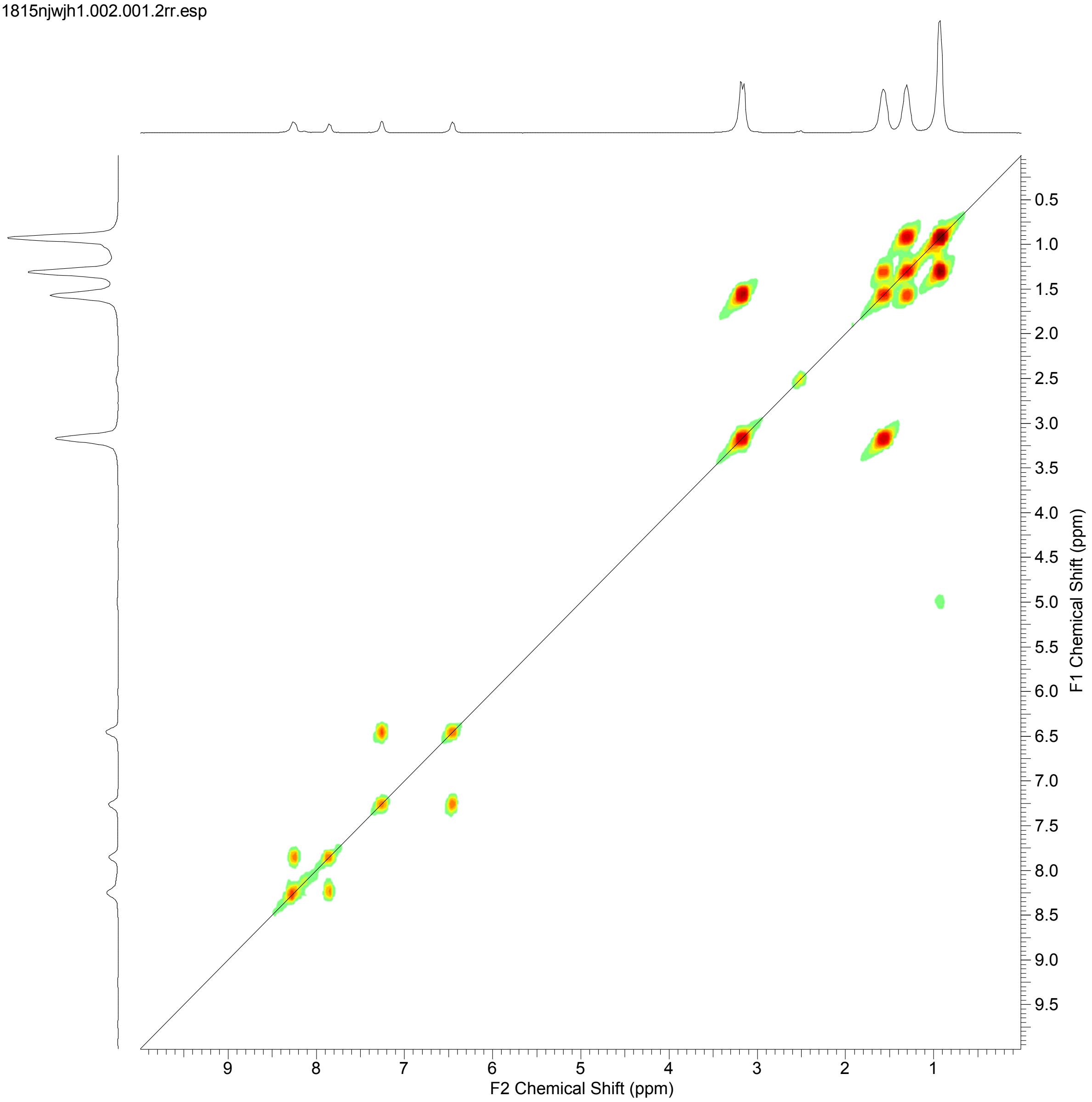

S52 ^{1}H COSY of an organogel (DMSO-d_6 (0.5 mL), compound **6** (80 mg) and compound **3** (11.5 mg)).

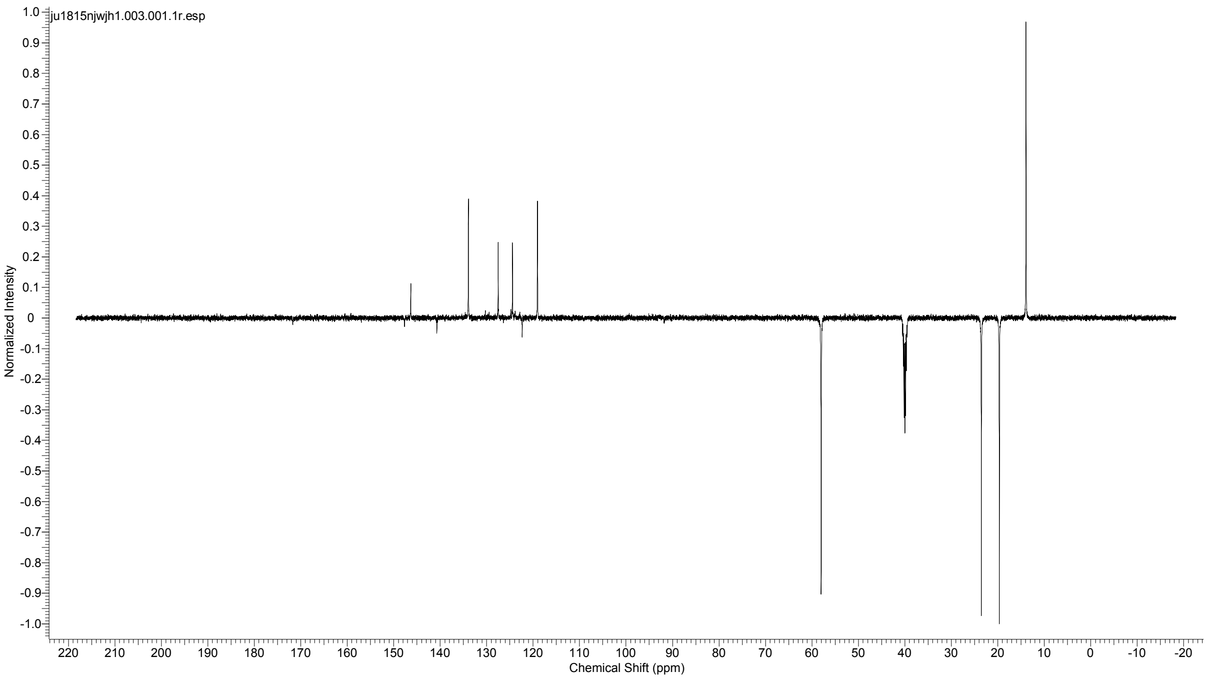

S53 JMOD ^{13}C NMR of an organogel (DMSO-d_6 (0.5 mL), compound **6** (80 mg) and compound **3** (11.5 mg)).

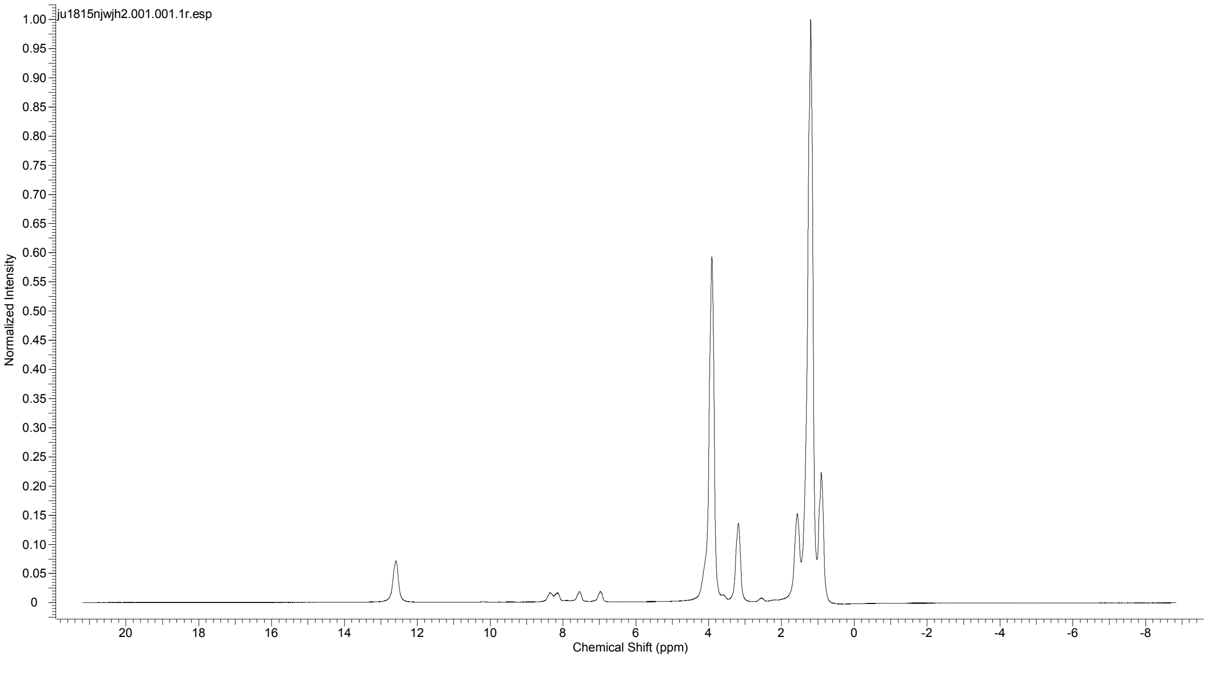

S54 ^{1}H NMR spectra of an organogel (DMSO-d_6 (0.5 mL), compound **6** (80 mg), DCP (0.1 mL) and compound **3** (11.5 mg)).

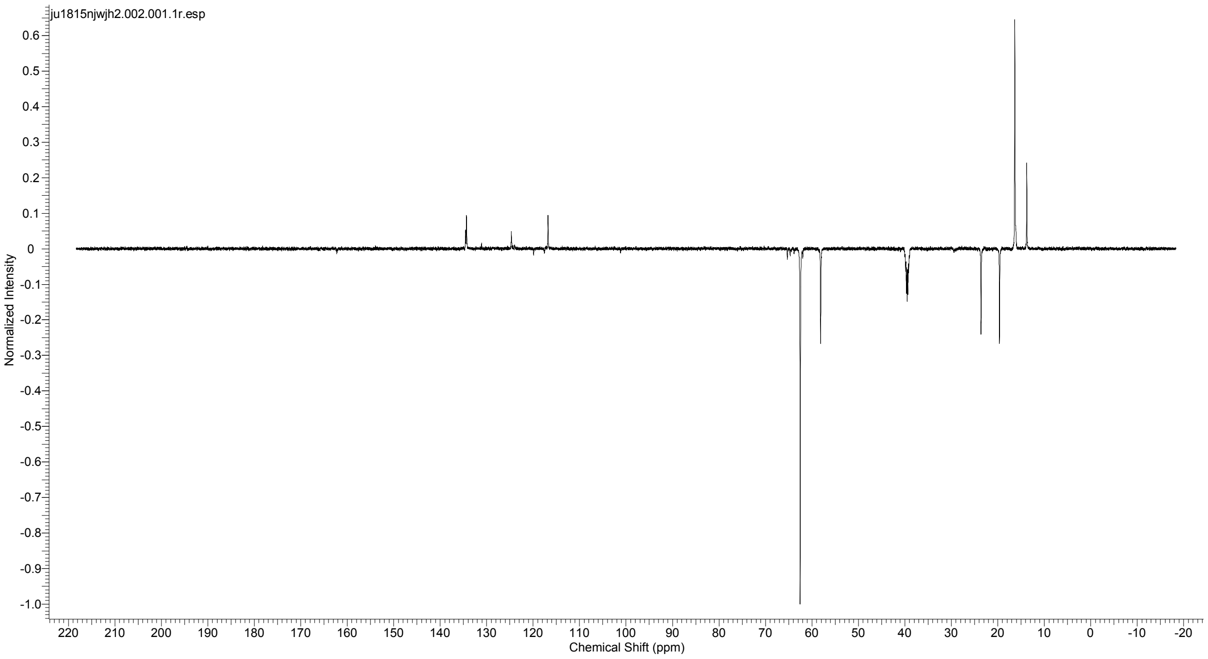

S55 JMOD ^{13}C NMR of an organogel (DMSO-d_6 (0.5 mL), compound **6** (80 mg), DCP (0.1 mL) and compound **3** (11.5 mg)).

References

1. J. R. Hiscock, I. L. Kirby, J. Herniman, G. J. Langley, A. J. Clark and P. A. Gale, *RSC Adv.*, 2014, **4**, 45517-45521.
2. H. Sato, T. Nakae, K. Morimoto and K. Tamura, *Org. Biomol. Chem.*, 2012, **10**, 1581-1586.
3. N. Zweep, A. Hopkinson, A. Meetsma, W. R. Browne, B. L. Feringa and J. H. van Esch, *Langmuir*, 2009, **25**, 8802-8809.
4. M. Kimura, T. Kitamura, T. Muto, K. Hanabusa, H. Shirai and N. Kobayashi, *Chem. Lett.*, 2000, 1088-1089.
